# How to improve photodynamic therapy-induced antitumor immunity for cancer treatment?

**DOI:** 10.7150/thno.72465

**Published:** 2022-05-29

**Authors:** Min Zhang, Yifan Zhao, He Ma, Yusong Sun, Jie Cao

**Affiliations:** 1 Department of Pharmaceutics, School of Pharmacy, Qingdao University, Qingdao 266021, China; 2 Institute of Biomedical Materials and Engineering, College of Materials Sciences and Engineering, Qingdao University, Qingdao 266071, China

**Keywords:** Photodynamic therapy, Antitumor immunity, Tumor microenvironment, Tumor vaccine, Synergistic therapies

## Abstract

Photodynamic therapy (PDT) is a promising method of tumor ablation and function-preserving oncological intervention, which is minimally invasive, repeatable, and has excellent function and cosmetic effect, with no cumulative toxicity. More importantly, PDT can induce immunogenic cell death and local inflammation, thus stimulating the body's immune response. However, the weak immunity induced by PDT alone is insufficient to trigger a systemic immune response towards cancer cells. To overcome this obstacle, multiple strategies have been investigated, including tumor microenvironment remodeling, tumor vaccines, subcellular-targeted PDT, and synergistic therapies. This review summarizes the latest progress in the development of strategies to improve the PDT-induced immune effect for enhanced cancer treatment.

## 1. Introduction

**Figure 1:**
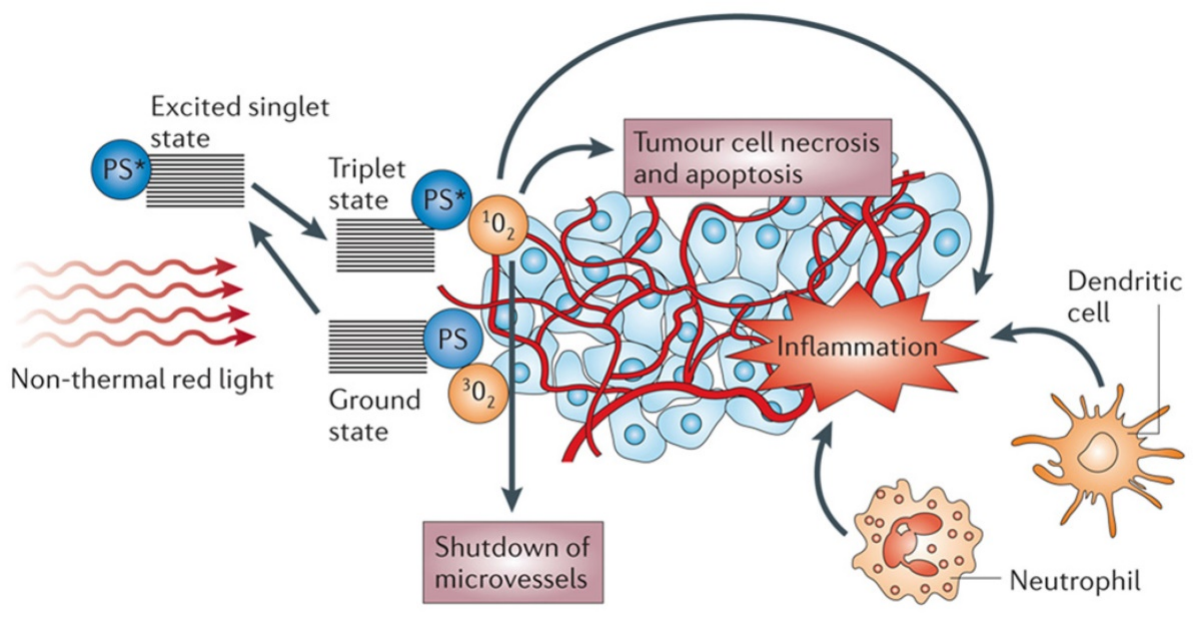
The mechanism of action on tumors in photodynamic therapy. Adapted with permission from 9, copyright 2006 Springer Nature.

Photodynamic therapy (PDT) is minimally invasive, with excellent function and no cumulative toxicity. PDT has been approved by the US Food and Drug Administration for the clinical treatment of various malignant tumors, including breast cancer, bladder cancer, and esophageal cancer 1-3. PDT has attracted increasing interest since Dougherty et al. 4 first demonstrated its application in 1975. PDT involves the local injection of a photosensitizer (PS) and local irradiation of the tumor with a specific wavelength of light to activate the PS. Subsequently, the excited PS transfers its energy to O_2_, resulting in cytotoxic reactive oxygen species (ROS), such as singlet oxygen (^1^O_2_), which can oxidize key cellular macromolecules and lead to tumor cell ablation 5, 6. PDT not only uses ROS to kill tumor cells and target tumor blood vessels but also activates the immune system to induce inflammation and immune response to tumor cells 7-10 (Figure 1).

It has been widely accepted that PDT can effectively stimulate both innate and adaptive immunity by immunogenic cell death (ICD). This form of cell death releases damage-associated molecular patterns (DAMPs), including calreticulin (CRT), high mobility group box 1 protein (HMGB1), adenosine triphosphate (ATP), and heat shock proteins (HSPs) 11, 12, leading to activation of the immune system, particularly by inducing the maturation of antigen presenting cells (APCs), which eventually migrate to lymph nodes, where cross-presentation of antigens to immature T cells triggers differentiation into cytotoxic CD8^+^ T cells. In addition, cytokines, such as interferon-γ (INF-γ), tumor necrosis factor-α (TNF-α), and interleukins (such as IL-2, IL-6, and IL-12), have also been reported to increase and contribute to immune stimulation 13. Therefore, photodynamically-induced ICD can activate specific immunogenicity and improve the therapeutic effect.

However, in most cases, PDT alone is insufficient to induce an adequate immune response as tumor cells also secrete immunosuppressive cytokines or other tumor-promoting molecules through non-immunogenic pathways, resulting in an immune-suppressive microenvironment, which inhibits the anticancer immune response. Hence, many studies have focused on improving the PDT-induced immune response, and have made significant progress. For instance, PDT combined with angiogenesis inhibitors can overcome the immunosuppression caused by hypoxia 14, while PDT combined with drug-targeted immunosuppression directly destroys the structure of the tumor microenvironment (TME) to improve PDT-induced anti-cancer immunity 15. Furthermore, in situ vaccines can be used to induce repeated cycles of immune initiation-immune effect-tumor cell death-antigen release, which can lead to an immune restart-immune effect in the local tumor to maximize the effect of anti-tumor immunity 16.

Recently, with the development of tumor immunology, some scholars have put forward the concepts of “cold” and “hot” tumors, which have gained attention in the context of anti-tumor immune research 17-19. The terms “hot” and “cold” have been commonly used to refer to T-cell infiltrating, inflamed but uninfiltrating, and uninflamed tumors. In the simplest terms, the so-called “cold” tumor is a non-immunogenic tumor with immunosuppression and insufficient T cell infiltration, while the “hot” tumor is the opposite. “Cold” tumors are good at camouflaging themselves from the immune system, thereby reducing the effectiveness of the immune response, which brings great obstacles to anti-tumor immunotherapy. Regulating the immune process to transform “cold” tumors into “hot” tumors has become a research hotspot in anti-tumor immunotherapy. Since researchers first reported that ICD can stimulate anti-tumor immunity, PDT-induced ICD has represented an attractive method to treat “cold” tumors. It is worth mentioning that the immune checkpoints have been shown to have a great prospect in cancer treatment, and their combination with PDT improves the prognosis of cancer and provides better control and effect for anti-tumor immunity 20. Additionally, PDT combined with other methods (such as photothermal therapy (PTT) 6, 21, chemotherapy 22-24, and radiotherapy 25) can better increase the recruitment of dendritic cells (DCs), promote the development of CD8^+^ T cells, regulate the TME, and activate host immune function.

**Figure 2:**
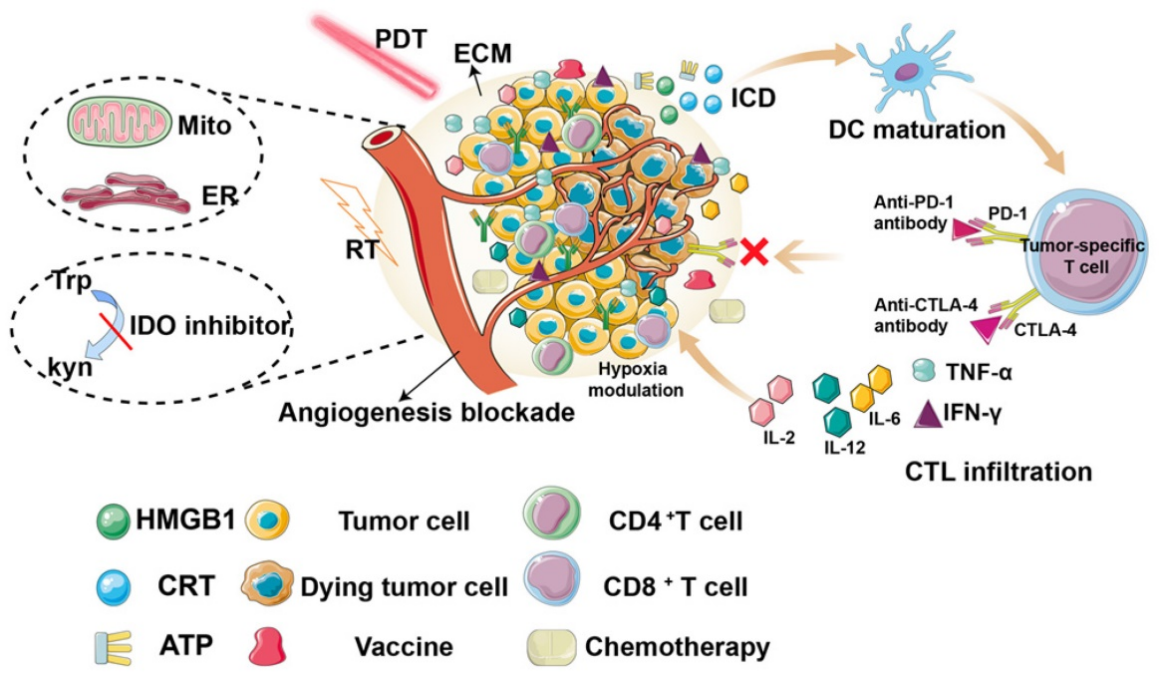
Overview of the strategies for improving photodynamic therapy-induced antitumor immunity for cancer treatment, including hypoxia modulation, ECM destruction, angiogenesis blockade, subcellular targeted, tumor vaccine construction, synergistic therapies.

Therefore, a comprehensive and in-depth depiction of the recent development of PDT-induced immune response is desirable. Compared to other current reviews, which mainly focus on nanoparticles 26, or a single aspect (such as TME 27, mitochondria 28), and with the further study of anti-tumor immune mechanisms, tumor vaccines have become the focus of immunotherapy research. However, to the best of our knowledge, there are few studies on the relationship between PDT and tumor vaccines, and no related review has been published. Thus, in this review, we comprehensively summarize the recent research on improving PDT-induced anti-tumor immunity, including reprogramming the TME, constructing tumor vaccines, and synergistic therapies (Figure 2, Table 1).

## 2. Reprogramming the TME

The host immune system determines the fate of tumor progression. Therefore, manipulating the immune system to activate the host immune response represents a promising strategy to develop an effective cancer treatment. Understanding the TME is critical to enhance the response of patients to immunotherapy. The TME is composed of cancer cells, various stromal cells (including fibroblasts, immune cells, endothelial cells, bone marrow-derived immature cells), cytokines (i.e., TNF, VEGF, IL-1), and chemokines (i.e., CXCL12, CCL27, CCL21) 29, 30. Cancer cells do not “work” alone, but interact closely with the extracellular matrix, stromal cells, and immune cells to promote chronic inflammation and immunosuppression.

Theoretically, treatment methods that act directly on tumor cells have many shortcomings, such as heterogeneity between tumors and tumor cells within tumor tissues, and instability of the biological or genetic characteristics of tumor cells. Therapeutic strategies targeting the TME have advantages, such as the fact that tumor interstitial cells have a stable genetic background and are not prone to mutation and drug resistance, the heterogeneity of the TME is reduced, and the curative effect is relatively stable. It is possible to predict the response of tumor tissue to treatment, which can play a critical role in controlling tumor metastasis 31.

As the importance of the TME in tumor progression and treatment is recognized, research into the improvement of PDT-mediated anti-tumor immunity by remodeling the TME is increasing 32-34 (Table 2).

### 2.1 Disrupting the tumor extracellular matrix (ECM)

The ECM is the “soil” of cells, and its topology and composition affect cell proliferation and differentiation. The ECM is a dense protein network surrounding normal and cancer cells, which forms an obstacle to drug delivery and seriously limits the penetration of immunosuppressants and other antineoplastic agents and the infiltration ability of immune cells to tumor sites 35. Therefore, the destruction of the ECM may represent an ideal way in which to improve PDT-induced anti-cancer immunity.

**Figure 3:**
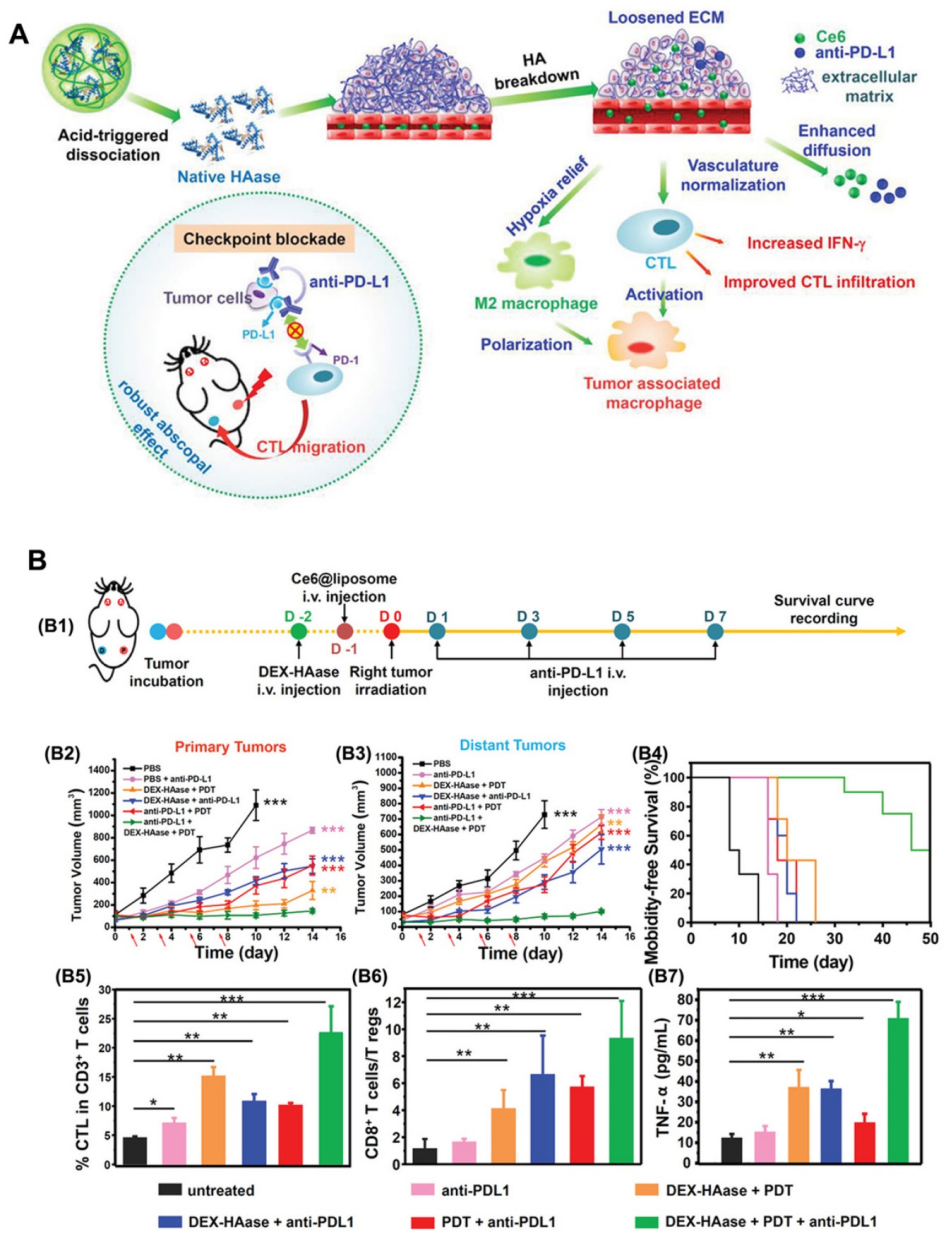
The mechanism of (A) DEX-HAase adjuvant and PD-L1 checkpoint enhancing PDT and anti-tumor immune response. (B) Evaluation of anti-tumor immunity in vivo. (B1) Schematic illustration of enhanced PDT and anti-PD-L1 combination therapy. In vivo results of (B2) primary and (B3) distant tumors growth curves of different groups of mice after various treatments. (B4) Morbidity-free survival of different groups of mice. (B5) CTL infiltration in tumors and (B6) the ratio of CD8^+^ T cells to regulatory T cells of mice post various treatments. B7) The production of TNF-α in serum of mice post various treatments. Adapted with permission from 37, copyright 2019 John Wiley and Sons.

Hyaluronic acid (HA) is a major component of the tumor ECM. Hyaluronidase (HAase) can rapidly degrade the ECM of tumors, loosening the skeleton of the ECM, thus enhancing drug penetration and T lymphocyte infiltration at the tumor site, which helps improve the effect of tumor immunotherapy 36. In light of this, Wang et al. 37 developed a stimulus-responsive HAase release system by using the natural macromolecular dextran (DEX) as a carrier. In this research, HAase was bonded with DEX through acidic cleavable groups under mild reaction conditions to obtain DEX-HAase nanoparticles that are stable in the physiological environment and rapidly release HAase in the acidic tumor environment (Figure 3). Specifically, in the acidic TME, the released HAase can decompose HA and thus destroy the ECM, increasing the content of effective blood vessels and improving the hypoxic conditions. Additionally, the increased oxygen in the tumor not only improves the efficacy of subsequent PDT but also increases the infiltration of cytotoxic T lymphocytes after PDT, thus achieving enhanced anti-tumor immunity. Compared to the large area of hypoxia observed in the untreated group, the hypoxia-associated fluorescence and the positive signals of hypoxia-inducible factor (HIF-1α) in the DEX-HAase group were significantly decreased. Moreover, the results of in vivo administration demonstrated significantly increased expression of IFN-γ, as well as increased infiltration of macrophages and CD8^+^ T cells in the DEX-HAase group, suggesting that DEX-HAase+PDT combined therapy can enhance cellular immunity. A bilateral tumor mouse model study further confirmed that PDT could enhance the release of tumor-associated antigens and the activation of DCs induced by ICD, which served to promote the infiltration of mature T cells to the TME.

### 2.2 Angiogenesis blockade

The local overgrowth of tumor blood vessels causes destruction of the tumor vascular structure, and the rich matrix components, such as collagen and HA, in the tumor constitute the physical barrier of intra-tumoral infiltration and jointly limit the infiltration of immune cells. Furthermore, the TME prevents immune cells, such as CD4^+^ and CD8^+^ T cells, from infiltrating the tumor site. Indeed, blocking tumor blood vessels can effectively inhibit tumor growth and prevent metastasis 38-40.

**Figure 4:**
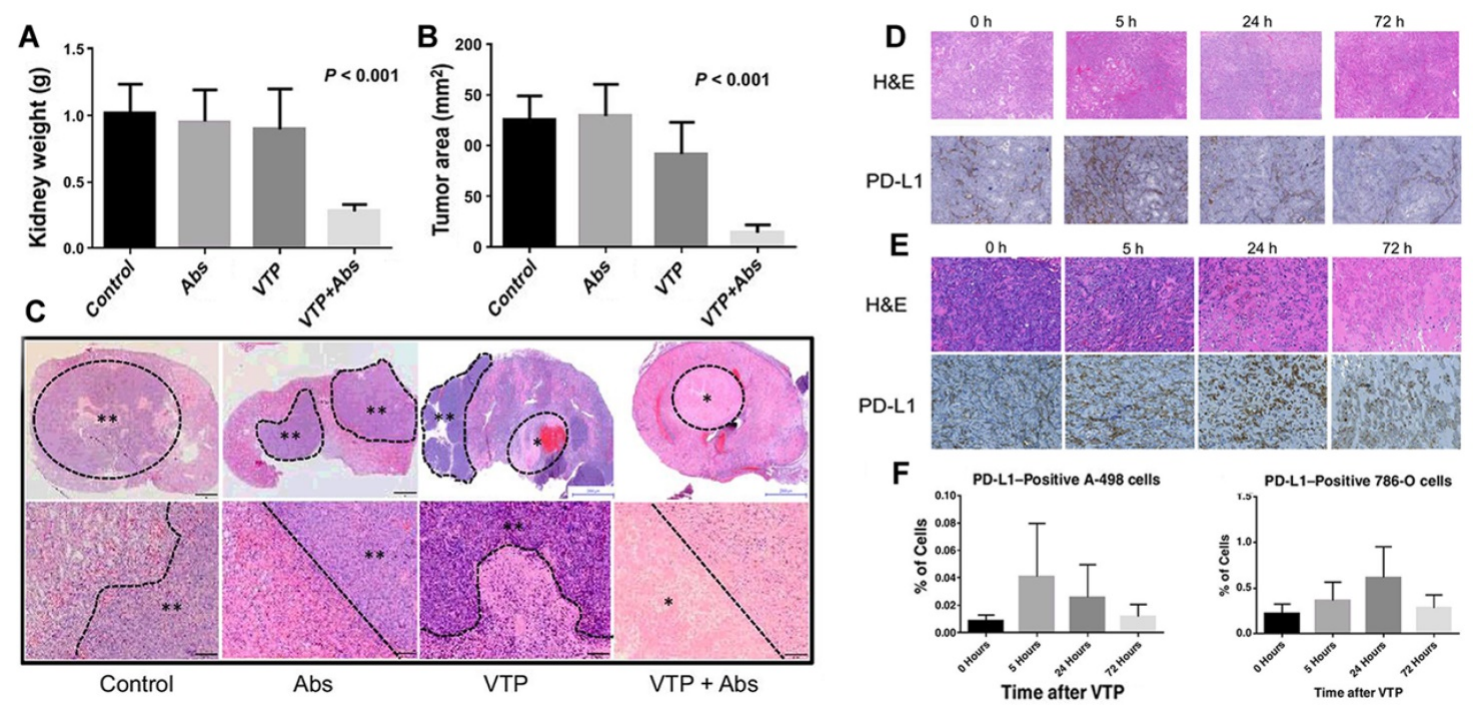
VTP and PD-1/PD-L1 blockade synergize to reject orthotopic renal tumors and prolong survival. On the 21st day, renal tumor growth was evaluated by renal weight (A) and maximum cross-sectional tumor area (B); (C) The kidneys were collected on the 21st day and stained with H&E. PD-L1 expression induced by VTP in (D) A-498 tumors and (E) 786-O tumors in the flanks of nude mice; (F) The ratio of PD-L1 positive cells in Amur498 tumor and 786muro tumor was evaluated. Adapted with permission from 43, copyright 2018 American Association for Cancer Research.

Vascular targeting PDT with the photosensitizer WST11 (TOOKAD-VTP) has been used clinically for treating early antecedent adenocarcinoma 41, 42. The results of phase II clinical trials of vascular targeting PDT (VTP) hemi ablation in patients with prostate cancer showed that compared to the untreated population, patients receiving VTP semi-ablation therapy had a high rate of no tumors after several years of treatment. O'Shaughnessy et al. 43 further studied the immunological potential of systematic anti-tumor treatment with TOOKAD-VTP in a mouse model of lung metastatic renal cell carcinoma. Primary tumors were treated with VTP, PD-1/PD-L1 antagonistic antibody (Abs), or combined therapy (VTP + Abs). The treatment response was observed, including immune infiltration of the primary and metastatic sites. As proposed in Figures 4A-C, compared to VTP or Abs alone, VTP combined with systemic PD-1/PD-L1 pathway block reduced the size of the primary tumor. Subsequently, histological analysis of the kidneys of mice receiving combined treatment showed no surviving tumor or necrosis. Moreover, comparing 17.5 days of VTP treatment and 19 days of Abs alone, the combined treatment group showed a prolonged survival time (27 days). The analysis of tumor-infiltrating lymphocytes showed that the therapeutic effect was related to the ratio of CD8^+^ Treg cells to CD4^+^FoxP3-Treg in the primary renal tumor, proving that VTP with the photosensitizer WST11 can induce a local immune response. More importantly, they found that VTP could induce PD-L1 expression in a human renal cell carcinoma xenograft model. As shown in Figures 4D-F, the proportion of cells expressing PD-L1 increased significantly after VTP treatment.

### 2.3 Response to tumor hypoxia

Hypoxia directly regulates the expression of various cytokines and affects the function of various immune cells, such as macrophages, APCs, and myeloid derived suppressive cells (MDSCs) 44. The heterogeneity of hypoxia can promote tumor invasion, metastasis, angiogenesis, and the increase in multidrug resistance proteins, all of which reduce the efficacy of anti-cancer drugs 45. Recently, various therapeutic strategies have been proposed for photodynamic hypoxia, including the delivery of oxygen to hypoxic tumors and the production of oxygen in hypoxic tumors 46-49.

#### 2.2.1 Delivering oxygen to hypoxic tumors

Using a suitable carrier to deliver O_2_ directly to the tumor is a commonly used method to overcome tumor hypoxia during PDT. Compared to the low loading efficiency of hemoglobin, the solubility of O_2_ in perfluorocarbon (PFC) is very high (approximately 40-50 mL O_2_/100 mL liquid, which is equivalent to the O_2_ solubility of 200 mL blood at 25°C and 1 atm). As a result, these substances have been used as key components in oxygen delivery systems 50, 51. For instance, Li et al. 22 synthesized a fluorinated cisplatin carrier (FS-PAMAM-Pt) and fluorinated photosensitizer Ce6 copolymer (F-Ce6-PEG), which could be assembled into nanoclusters FS@PMPt by interacting fluorine-fluorinated (FF) with PFC. Under laser irradiation, the oxygen in PFC ensured sufficient production of ROS to promote ICD-induced immune activation. Moreover, in the mouse breast 4T1 tumor model, the tumor growth treated with FS-PAMAM-Pt was inhibited, the tumor volume was decreased, the morphology was altered, and high rates of necrosis were observed. Flow cytometry detection of CD40 and CD86 on CD11c^+^ DCs showed that after FS@PMPt treatment, CD8^+^ T cell infiltration was increased, while the proportion of Tregs was decreased. Furthermore, the activation of the anti-tumor immune response was accompanied by the production of IFN-γ. Following treatment with the FS@PMPt laser, the production of IFN- γ was increased by 4.4%, while the expression of TGF-β decreased by approximately 53%, which further proved the transformation from an immunosuppressive “cold” tumor to a “hot” tumor and enhanced photodynamic immunotherapy.

**Figure 5:**
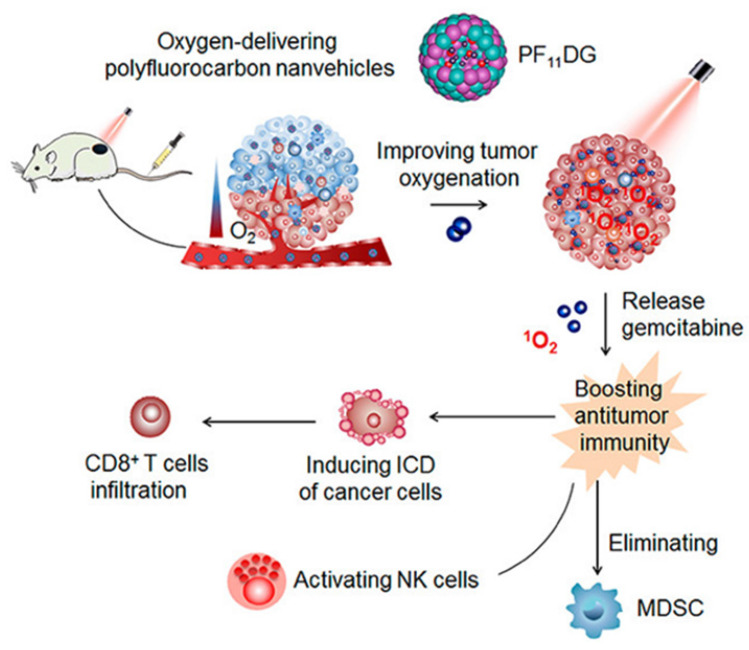
Schematic illustration of mechanism of PF11DG to enhance tumor oxygenation and elicit antitumor immune responses for cancer therapy. Adapted with permission from 52, copyright 2021 American Chemical Society.

However, the FS@PMPt particle size of 165 nm may be associated with problems such as limited penetration of the tumor anoxic zone. Alternatively, fluorocarbon chains can be chemically linked or polymerized into polyhedral amphiphilic polymers and further assembled into nanocarriers, which have been used to promote cell membrane penetration or tumor-targeted biomedical imaging. Therefore, Wang et al. 52 reported the ability of an amphiphilic oxygen-supplied polyfluorocarbon nanocarrier loaded with the photosensitizer DiIC_18_ (5) (DiD) and the gemcitabine prodrug (named as PF_11_DG) to relieve tumor hypoxia and enhance anti-tumor immunotherapy. DiD as a photosensitizer can effectively produce ROS in tumor cells and induce the occurrence of ICD, while gemcitabine can enhance anti-tumor immunity through activating the cytotoxicity of natural killer cells and eliminating immunosuppressive MDSCs in the tumor. (Figure 5). In tumors, PF_11_DG showed flexible intratumoral infiltration under laser irradiation, which enhanced the strong anti-tumor immune response. It is worth noting that PF_11_DG plus laser irradiation (PF_11_DG+L) significantly delayed tumor growth, with an inhibition rate of 82.96% in the 4T1 breast cancer model and 93.6% in the PANC_02_ pancreatic cancer model.

#### 2.2.2 Producing oxygen in a hypoxic tumor

Due to metabolic abnormalities, cancer cells produce higher levels of hydrogen peroxide than normal cells (concentrations ranging from 10^-4^ to 10^-3^ M) 53. Therefore, the design of compounds that can degrade endogenous H_2_O_2_ to produce O_2_ in tumors is one way to alleviate the level of hypoxia.

**Figure 6:**
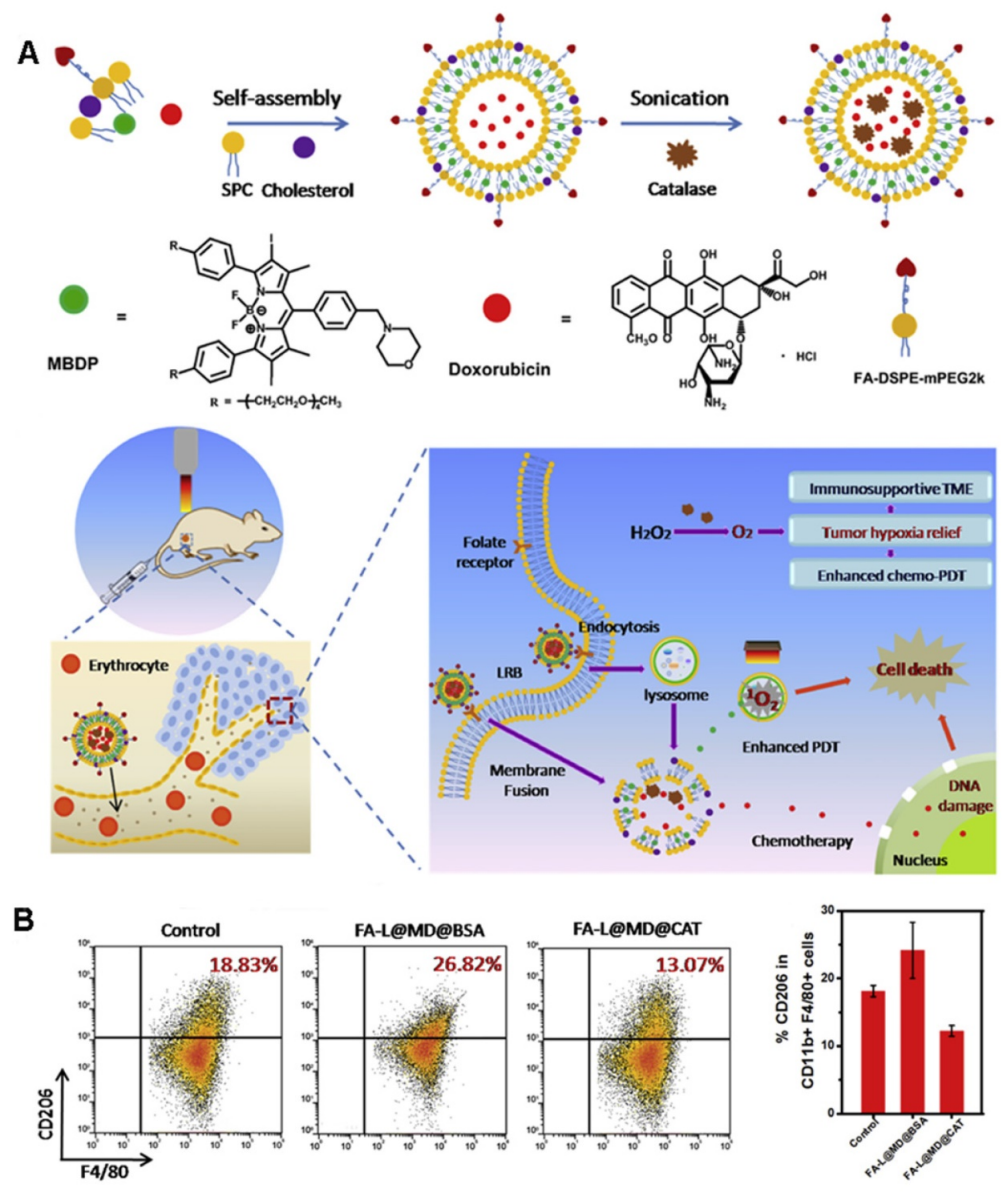
(A) The preparation scheme and mechanism of FA-L@MD@CAT to Enhance Antitumor Immune Responses for Cancer Therapy. (B) Representative flow cytometer plots and the corresponding quantification of M2-type macrophages (CD206^+^). Adapted with permission from 54, copyright 2020 Elsevier.

Shi et al. 54 developed a unique liposome FA-L@MD@CAT to encapsulate catalase (CAT), a photosensitizer (MBDP), and adriamycin to catalyze the overexpression of H_2_O_2_ in tumors and increase tumor oxygenation, thereby reversing the immunosuppressive TME (Figure 6A). Under 660-nm irradiation, FA-L@MD@CAT can greatly promote the production of^ 1^O_2_. Most importantly, under the condition of hypoxia, the polarization of macrophages changes from the anti-tumor M1 phenotype to the tumor-promoting M2 phenotype 55. As shown in Figure 6B, the proportion of M2-type macrophages in the FA-L@MD@CAT treatment group was significantly lower than that in the control group. Similarly, a significant increase in the proportion of cytotoxic T lymphocyte (CTLs) was detected by flow cytometry. Therefore, the enhancement of tumor oxygenation significantly induces tumor cell death by regulating the expression of immune cytokines, reversing the immunosuppressive TME, and promoting the anti-tumor immune response.

In addition to enzymes that can catalyze H_2_O_2_, metal-based oxygen generators such as manganese dioxide (MnO_2_), calcium oxide, cerium oxide (CeO_2_), and titanium oxide (TiO_2_) can catalyze H_2_O_2_ to generate O_2_, thereby relieving tumor hypoxia, enhancing PDT, and further enhancing anti-tumor immunity.

**Figure 7:**
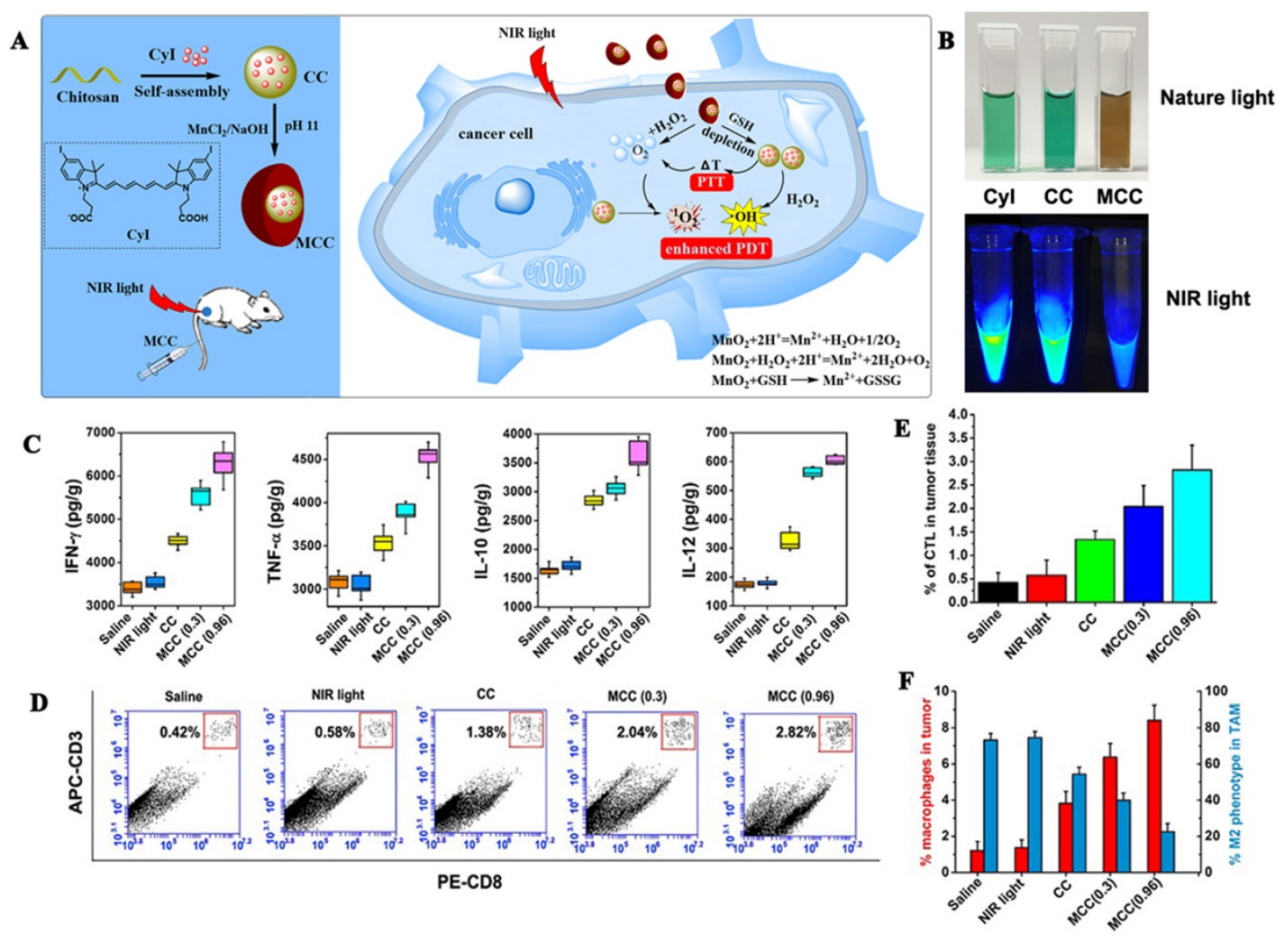
(A) Activation mechanism of MCC nanosystems for highly efficient phototherapy and acute immune response; (B) Photos of CyI, CC, and MCC in water solution under ambient light and NIR light; (C)The immune factor concentrations of IFN-γ, TNF-α, IL-10, IL-12; (D) Flow cytometry data of cytotoxic T lymphocyte (CTL) infiltration in tumors. CD3^+^ and CD8^+^ T cells were defined as CTLs; (E) CTL quantification of flow cytometry results; (F) Macrophage infiltration and polarization within tumors after various treatment. Adapted with permission from 56, copyright 2020 Ivyspring.

MnO_2_ catalyzes the decomposition of H_2_O_2_ to O_2_ under neutral conditions and reacts with H^+^ to O_2_ under acidic conditions. An advantage of MnO_2_ is that it can adsorb different types of small molecules by physical adsorption or Mn-N coordination bonds. Our research group 56 used manganese dioxide to design a MnO_2_@chitosan-CyI (MCC) dual oxygen production nanosystem to enhance the combined therapy of tumors (Figures 7A, B). In this experiment, self-assembly of the iodinated indocyanine green (ICG) derivative CyI and chitosan was conducted to prepare a TME-sensitive nanosystem, following which, a shell of MnO_2_ nanoparticles was constructed on the surface through electrostatic interactions, and Mn-N coordination bonds. Under the irradiation of near-infrared light, MCC can enhance the production of ROS and heat. Additionally, once the material is internalized by the cells, MnO_2_ can be used as an efficient in situ oxygen generator to alleviate hypoxia in the TME. Meanwhile, the heat generated by the nanosystem can also increase the temperature and accelerate the blood flow in the body, thereby further alleviating hypoxia. Additionally, as shown in Figures 7C-D, enhanced PDT can also trigger an acute immune response, and the resulting combination of PDT-photothermal-immune therapy can effectively eliminate the primary tumor and suppress tumor metastasis.

**Figure 8:**
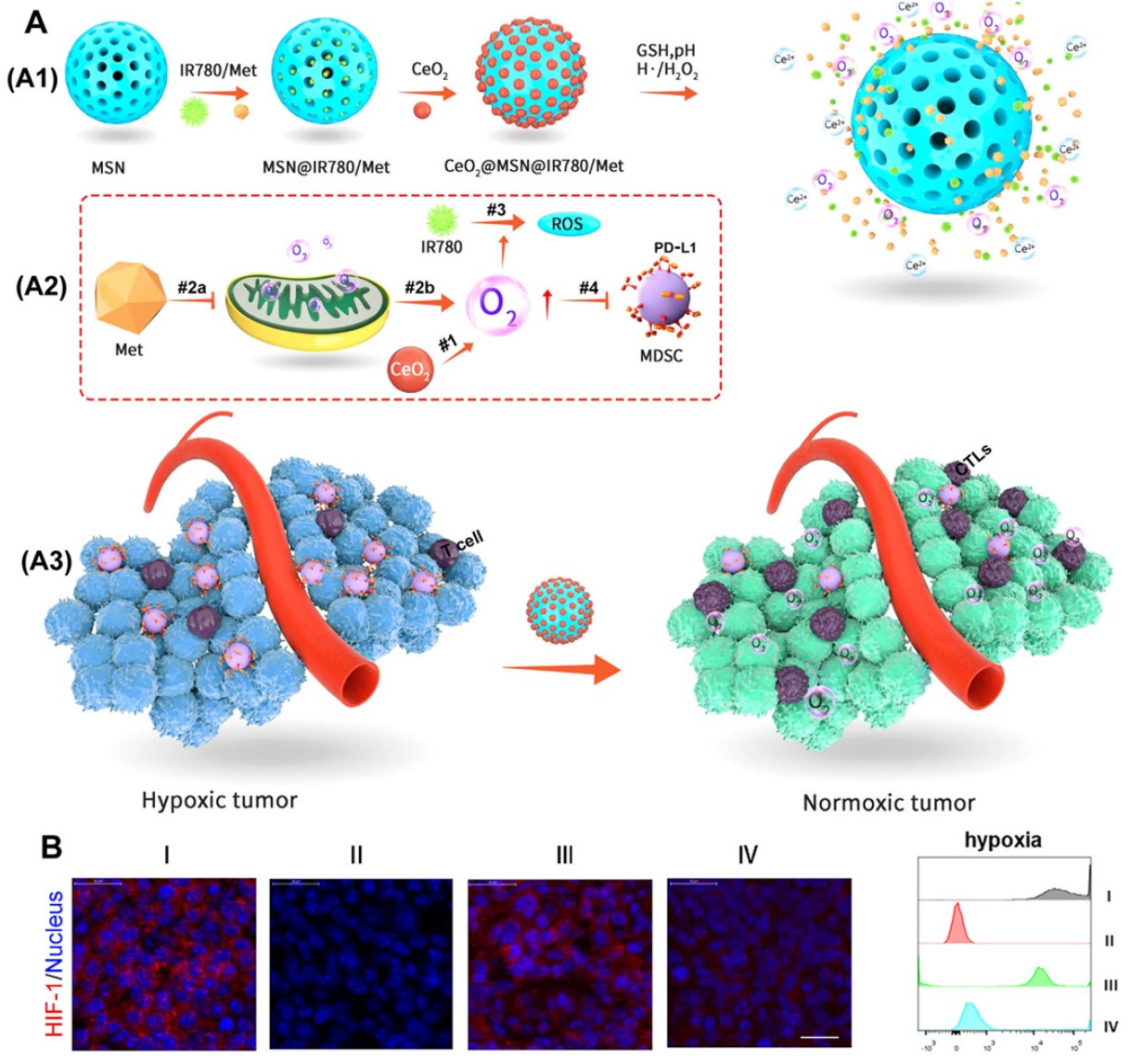
(A) Working principle of CeO_2_@MSNs@IR780/Met NPs. (A1) CeO2@MSNs@IR780/Met NPs were synthesized by loading IR780 and Met into MSNs with CeO_2_ as the gatekeepers. (A2) Schematic diagram of the CeO2@MSNs@IR780/Met NPs for enhanced PDT. (A3) Application of CeO2@MSNs@IR780/Met NPs markedly activated and strengthened the immune response; (B) Immunofluorescence images and FCM showing the HIF-1 expression in B16F10 cells after different treatments. Adapted with permission from 59, copyright 2020 American Chemical Society.

CeO_2_ has a similar chemical catalytic activity to peroxidase and oxidase and can convert H_2_O_2_ into highly toxic hydroxyl radicals under acidic tumor conditions 57, 58. Therefore, as proposed in Figure 8A, Zuo et al. 59 used the catalytic oxygen production ability of CeO_2_ to wrap it on mesoporous silica nanoparticles (MSNs) and load metformin (Met) and photosensitizer (IR780) inside to prepare an oxygen-replenished photodynamic nanoplatform (CeO_2_@MSNs@IR780/Met). CeO_2_ nanoparticles can catalyze the overexpression of H_2_O_2_ in tumor tissues to produce oxygen, while the inhibition of mitochondrial respiration by Met can cause the accumulation of O_2_ in the tumor and further reduce tumor hypoxia. Thus, this nanoplatform can be used to effectively modify the hypoxia of the TME (Figure 8B). More importantly, the nanoplatform can effectively recruit CTLs and significantly reduce the expression of PD-L1 on MDSCs, which together, greatly destroyed the immunosuppressive function of MDSCs and significantly enhanced the anti-tumor immune response of PDT.

## 3. Subcellular-targeted PDT

Recently, an increasing number of studies have shown that selective delivery of drugs to specific subcellular organelles can significantly influence the immunotherapeutic effects. Among them, the endoplasmic reticulum (ER) and mitochondria play an important role in regulating the apoptosis and metabolism of cancer cells and are more vulnerable to hyperthermia and oxidative damage. Therapeutic strategies targeting the endoplasmic reticulum (ER) and mitochondria have broad prospects in the field of PDT-induced immunotherapy.

### 3.1 Endoplasmic reticulum-targeted PDT

**Figure 9:**
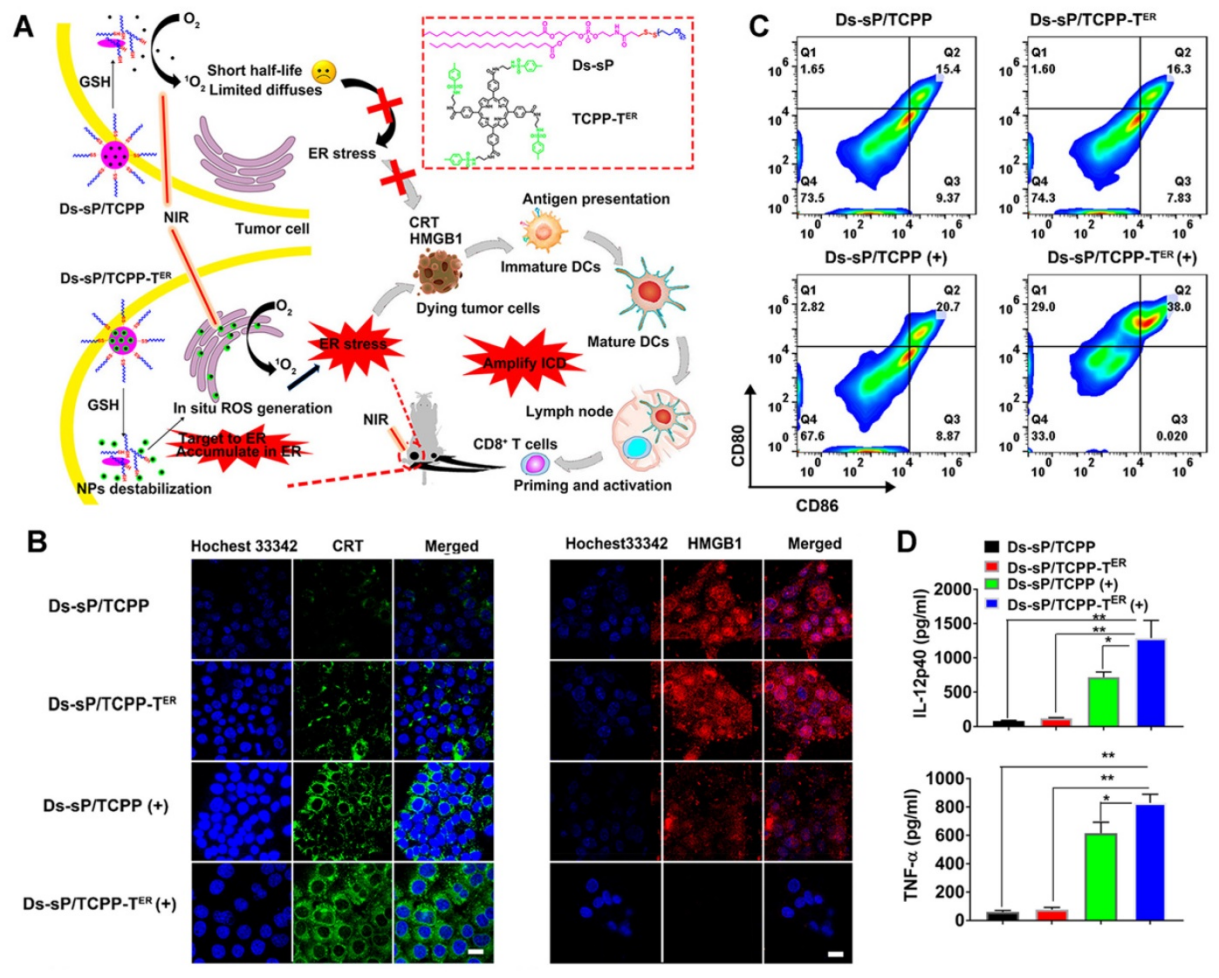
(A) Schematic diagram of the Ds-sP/TCPP-T^ER^ for enhanced PDT. (B) CLSM images showing surface translocation of CRT and HMGB1 release with Ds-sP/TCPP or Ds-sP/TCPP-T^ER^ with or without 670 nm laser irradiation. (C) 4T1 cells were treated with Ds-sP/TCPP or Ds-sP/TCPP-T^ER^ with or without 670 nm laser irradiation, followed by coculture with BMDCs. The BMDCs were stained with anti-CD86 and anti-CD80 antibodies and analyzed using flow cytometry. (D) Quantification of secretion of IL-12P40 and TNF-α in DC suspensions. Adapted with permission from 64, copyright 2020 American Chemical Society.

As a unique multifunctional organelle, ER plays a crucial role in maintaining intracellular signal transduction, calcium homeostasis, protein synthesis, and processing 60, 61. ROS production induces endoplasmic reticulum stress, which is considered a major cause of ICD 62, 63. Therefore, exploring the use of PDT for targeting the ER is an optimal scheme to effectively induce photodynamic immunity. For example, Deng et al. 64 synthesized reduction-sensitive nanoparticles, Ds-sP NPs, and uploaded an efficient ER targeting photosensitizer, TCPP-TER (Figure 9A). As proposed in Figures 9B-D, under NIR irradiation, Ds-sP/TCPP-T^ER^ NPs can selectively accumulate in the ER and produce ROS locally, which can induce ER stress, magnify ICD, and activate immune cells, thus enhancing the effect of immunotherapy. Both in vitro and in vivo studies have shown that the ER-targeting PDT strategy can enhance the ICD effect and promote the release of DMAPs. Additionally, the secretion of cytokines in the tumor site increased, as did the infiltration of CD8^+^ T cells, suggesting that this strategy can improve the efficiency of immunotherapy.

**Figure 10:**
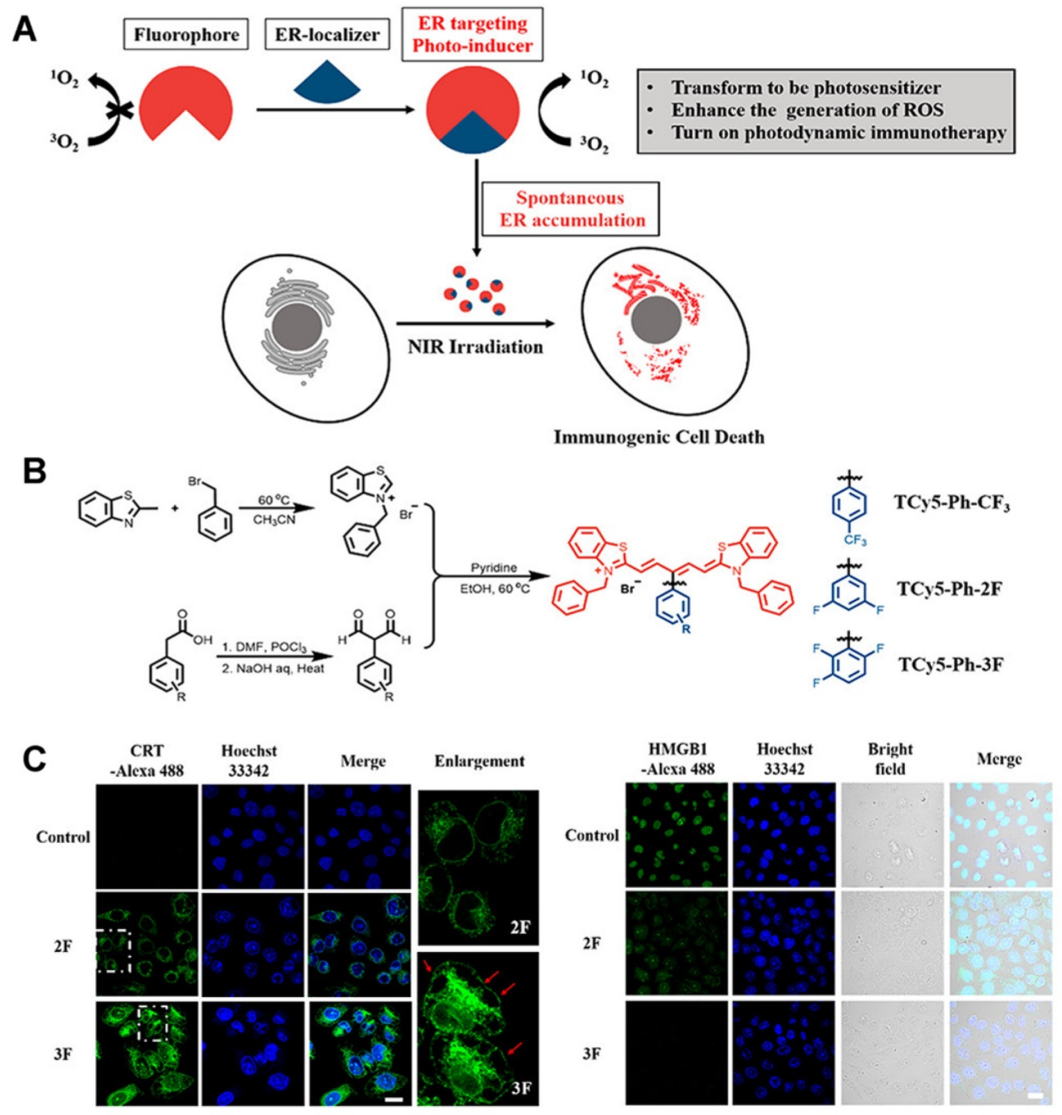
(A) Schematic of the ER localizer mediating precise construction of the photosensitizer for the ER. B) Schematic illustration of the synthesis of TCy5 derivatives. (C) Immunofluorescence staining images of calreticulin proteins, HMGB1-positive and extracellular release of ATP after different treatments. Adapted with permission from 65, copyright 2022 American Chemical Society.

The use of an efficient ROS generator targeting the ER is the best scheme to effectively induce endoplasmic reticulum stress. Thus, Ma et al. 65 developed the first thio-pentamethyne cyanine photosensitizer, TCy5-Ph-3F, which can be used as an ICD photoinducer (Figures 10A-B). Irrespective of normoxic or hypoxic conditions, TCy5-Ph-3F has a selective tendency toward ER accumulation and excellent ROS production ability (the singlet oxygen quantum yield [ΦΔ] is 39%). With the help of targeting ligands, the photosensitizer can be localized to the ER, and, under near-infrared radiation, exert a photodynamic effect, generate a large amount of ROS, and cause ER stress, triggering an immune response. Moreover, TCy5-Ph-3F could induce the production of many DMAPs, including up-regulation of glucose-regulated protein-78 (GRP-78), HMGB1 efflux, and secretion of ATP, CRT, and HSP70 (Figure 10C). The maturation of DCs and the activation of CD8^+^ T cells were also detected in vivo, indicating that TCy5-Ph-3F can activate the systemic immune response. These results can guide the design of an efficient ICD photoinducer and expand the application prospect of organic molecules in cancer immunotherapy.

### 3.2 Mitochondrial-targeted PDT

Mitochondria play an important role in determining cell fate and regulating cell physiological changes, signal transduction pathways, and metabolism 66, 67. More importantly, many studies have emphasized that mitochondria are essential for the metabolism and activation of immune cells 68-70. Indeed, increased mitochondrial biomass and reserve respiratory capacity have been shown to enhance T cell persistence and provide the necessary bioenergy advantages to eradicate cancer cells and prevent recurrence 71, 72. As the most sensitive subcellular organelle to ROS, mitochondrial-targeted PDT has been considered an effective strategy for anti-tumor immunotherapy.

Based on the principle that the inner mitochondrial membrane is highly negatively charged, the lipophilic cationic triphenylphosphine (TPP) targeting of mitochondria has been widely used. As the mitochondrial membrane potential of tumor cells is higher than that of normal cells, lipophilic cationic TPP as the mitochondrial targeting ligand can penetrate the mitochondrial membrane only by electrostatic adsorption, which greatly increases the aggregation of particles in the mitochondria of tumor cells 73-75.

**Figure 11:**
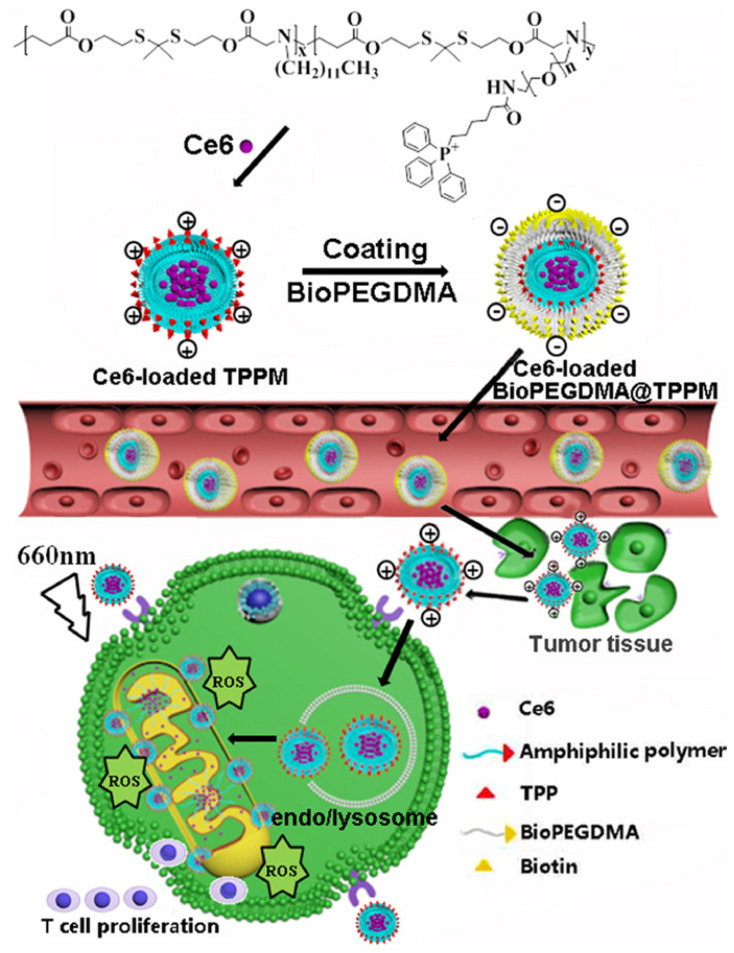
Schematic diagram of the composition and transportation process of BioPEGDMA@TPPM for enhanced PDT. Adapted with permission from 75, copyright 2020 Elsevier.

As illustrated in Figure 11, Peng et al. 75 successfully constructed a mitochondrial targeted delivery system. In this study, positively charged micelles loaded with amphiphilic copolymer Ce6 were used as the core and the biotin-PEG4000-NH_2_ modified by anionic 2, 3-dimethylmaleic anhydride (DMA) was used as the shell to form BioPEGDMA@TPPM. First, the biotin in the shell led the particles to target the tumor cells and accumulated effectively in the tumor tissue. Secondly, the shell falls off and the exposed TPP targets the mitochondria. Consequently, Ce6 induces ICD, which promotes the production of immune cytokines, thereby enhancing the immune response. The photodynamic antitumor activity and immune response of mitochondria in Kunming mice and BALB/C nude mice bearing CT26 cells were detected *in vivo*. Compared to other treatment groups, the Ce6-loaded BioPEGDMA@TPPM system showed a good inhibitory effect on primary tumors, with a tumor inhibition rate of 93.2% and 84.1% in Kunming mice and BALB/c mice, respectively. On the 14th day after PDT treatment, various immune signal molecules were detected in tumor and lymphoid tissues, including IFN-γ, TNF-α, and CD4^+^T cells, CD8^+^ T cells, and DCs. The results showed that the fluorescence intensity and activation percentage of CD4^+^ T cells, CD8^+^ T cells, and DCs were significantly increased in tumor tissues following treatment with the Ce6-loaded BioPEGDMA@TPPM system. Following accumulation in the mitochondria, this system effectively boosted the cellular immune response and assisted PDT to exert an anti-tumor immune response.

The concept of combining PDT with ICD has been proposed to overcome the problems of high tumor recurrence rates and tumor drug resistance, which cannot be solved by a single therapy. However, most previous studies focused on the ER-stress injury-related DAMPs, while ignoring the secretion and function of mitochondria-related DAMPs.

**Figure 12:**
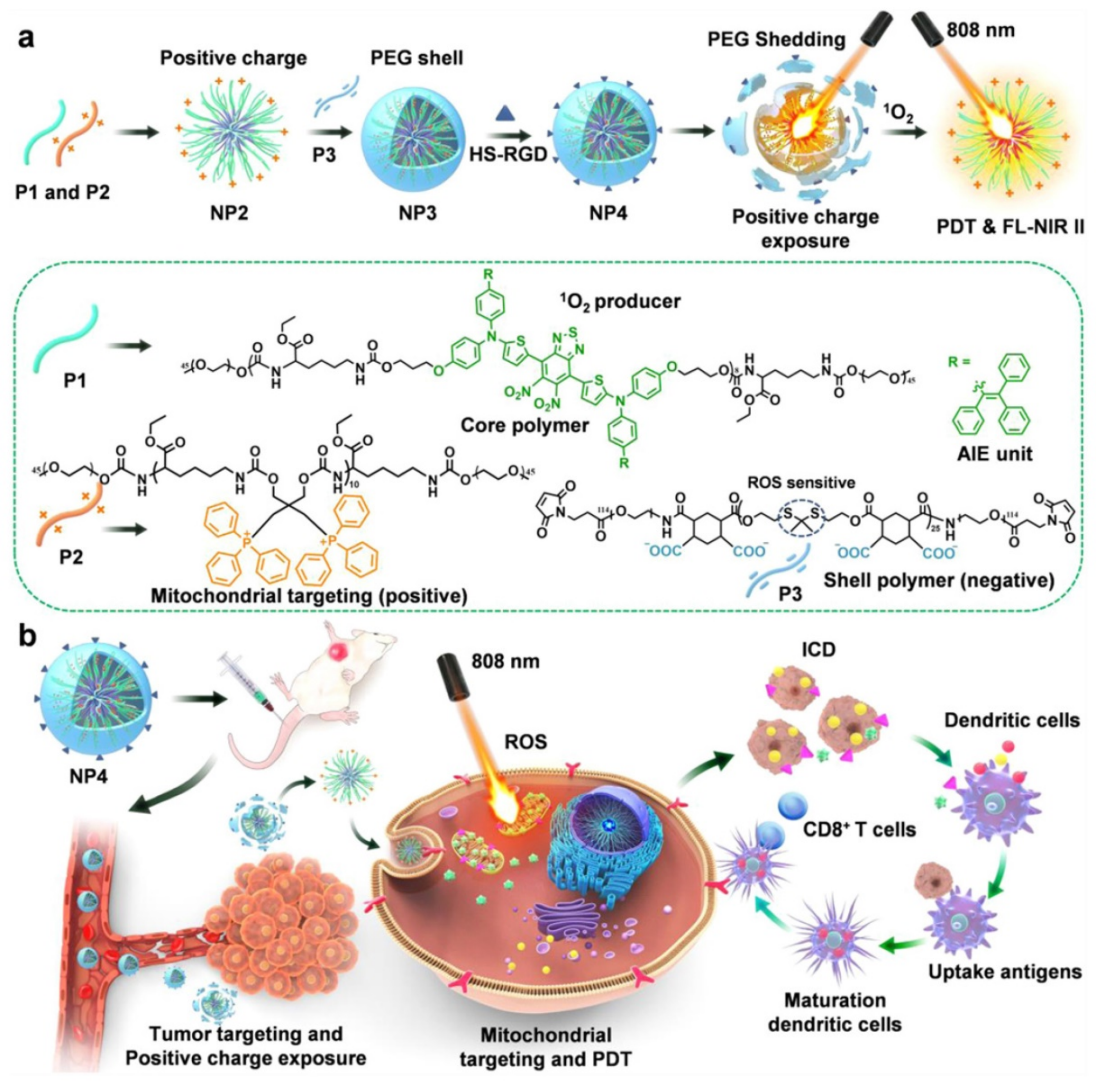
(A) Design of core shell nanoparticles and illustration with dual-cascade targeting (DCT) performance triggered by NIR light for maximizing the efficacy of photodynamic therapy (PDT) and cancer immunotherapy. Adapted with permission from 76, copyright 2021 Elsevier.

As illustrated in Figure 12A, Wei et al. 76 developed a nanodelivery system that can be controlled by NIR to achieve cascade targeting of cancer cells and immune cells. In this study, they synthesized three types of polymers by polycondensation, namely, degradable infrared two-zone fluorescent polymer P1, triphenylphosphine polymer P2 with mitochondrial targeting, and polymer P3, whose thione structure can be destroyed in the presence of ROS. The positively charged NP2 nanoparticles were prepared by co-assembly of P1 and P2, then NP2 and P3 were used to form NP3 by electrostatic adsorption. Finally, NP3 was combined with the targeting group HS-RGD to obtain NP4 nanoparticles with cascade targeting ability. To verify the biological effects of the nanoparticles, they constructed an in situ tumor model of triple-negative breast cancer 4T1 cells. NP4 injected into mice through the tail vein was shown to pass through the blood circulation before accumulating at the tumor site. At the tumor site, NP4s produce ROS under the excitation of an 808-nm laser, which makes the PEG on the surface of NP4 fall off to generate NP2 nanoparticles, thus realizing charge reversal. NP2 can further penetrate the tumor tissue and target the mitochondria in the tumor cells. As shown in Figure 12B, under continuous light, NP2s produce a large amount of ROS in mitochondria, which can kill cancer cells. Simultaneously, DAMPs induced by PDT promote the maturation of DCs, and mature DCs present antigens to T cells, thus inducing CD8^+^ T cell differentiation and activating adaptive immunity, thereby realizing the combined therapy of PDT and host immunity.

## 4. PDT combined with Immunotherapy

Immunotherapy enhances the ability of the immune system to target cancer cells and has shown efficacy against various tumors. The local stimulation induced by PDT is insufficient to induce an effective immune response. Therefore, the combination of PDT with immunotherapy has become a promising choice in recent years 20, 77, 78. In this section, we will summarize the PDT combination immunotherapy (Table 3).

### 4.1 Tumor vaccines

As a promising method of cancer immunotherapy, therapeutic tumor vaccines have become a powerful weapon for treating cancer. Tumor vaccines refer to the use of a tumor antigen, through active immunity, to induce the body to produce specific anti-tumor effects stimulate the body's immune protection mechanism, treat tumors or prevent recurrence. PDT can release tumor-associated antigens (TAAs) through local selective photochemistry, induce ICD, enhance antigen presentation, and activate in situ immune cells (such as T cells) to form an in situ tumor vaccine. However, the residual tumor after PDT may be insufficient to independently induce an effective anti-tumor response. Consequently, additional immune stimulation is often needed to induce the immune system to respond to residual tumor fragments or metastatic tumor cells, including immune adjuvants 79, 80 and immune cells 81, 82, 20, 83.

#### 4.1.1 Adjuvant

An adjuvant, also known as an immune potentiator, is a vaccine additive that, when injected into the body before or mixed with antigen, can enhance the immune response to antigen or change the type of immune response. Adjuvants serve as non-specific immune enhancers but have no antigenicity 84.

**Figure 13:**
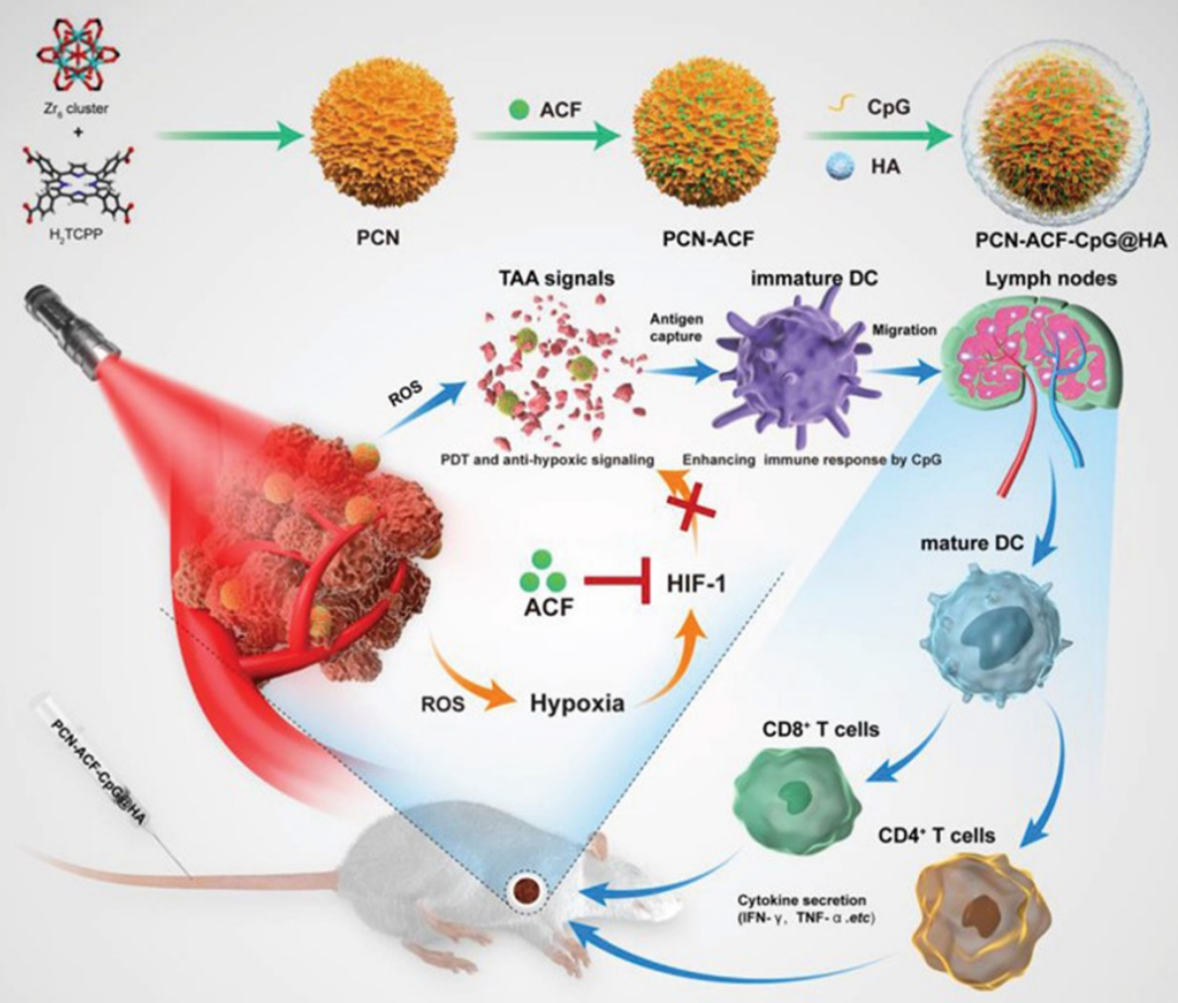
Schematic illustration of the preparation procedure and the working principle of PCN‐ACF‐CpG@HA to integrate PDT, antihypoxic signaling, and CpG adjuvant as in situ tumor vaccine. Adapted with permission from 85, copyright 2020 John Wiley and Sons.

For instance, the adjuvant CpG can directly activate B cells and DCs to produce an environment rich in pro-inflammatory factors and Th1 cells. CpG can promote the development of CTL through IFN-c induced T cells and increase the production of IL-6 and IL-12 to promote antibody secretion 84. Accordingly, Cai et al. 85 designed and synthesized nanoparticles based on a metal-organic framework, which combined PDT and antioxidant signal transduction with a CpG adjuvant as an in situ tumor vaccine to enhance the PDT-induced anticancer response (Figure 13). The results showed a strong ROS signal after PDT of cancer cells, specifically targeting the CD44 receptor overexpressed by HA, while Acriflavine blocked the expression of HIF-1α. With the aid of CpG, TAAs produced by PDT-induced tumor cell damage can enhance the presentation ability of DCs, which promotes the maturation of CD4^+^T cells and CD8^+^ T cells and the release of cytokines (including INF-γ, TNF-α). Taken together, these novel in situ immunostimulatory strategies can enhance the anti-tumor effect of PDT by activating the host anti-tumor immune response *in vivo* and *in vitro*.

Nanodrugs have great potential to provide safer and more effective cancer immunotherapies by improving the pharmacokinetics and biodistribution of immunotherapeutic drugs and enhancing their interaction with immune cells. Additionally, nanomedicine can provide multiple treatments in one preparation and work in conjunction with immunotherapy to develop more effective anti-tumor combination therapies. Therefore, endogenous trigger-sensitive nanodrugs show great potential in the context of anti-tumor immunity in a controlled manner for the accurate delivery of immunotherapeutic agents.

Liu et al. 86 synthesized an intelligent semiconducting polymer nanoimmunomodulator (SPNI) for acid TME-activated precision photodynamic immunotherapy using endogenous and exogenous triggers. Through the coupling of an acid-labile Schiff-based linker with Toll-like receptor 7 (TLR7) agonists, R837 was introduced to trigger TLR7 junctions located in the endomembrane to promote DC maturation and pro-inflammatory cytokine secretion. After accumulation in the tumor site, SPNI is hydrolyzed and limits the specific release of R837 in response to the acidic environment in the tumor tissue. When exposed to near-infrared light, SPNI exerts a photodynamic effect to mediate direct tumor ablation and ICD production. The released immunogenic factors and the precise activation of the tumor TLR7 pathway trigger adaptive anti-tumor responses through an in situ vaccine-like function.

**Figure 14:**
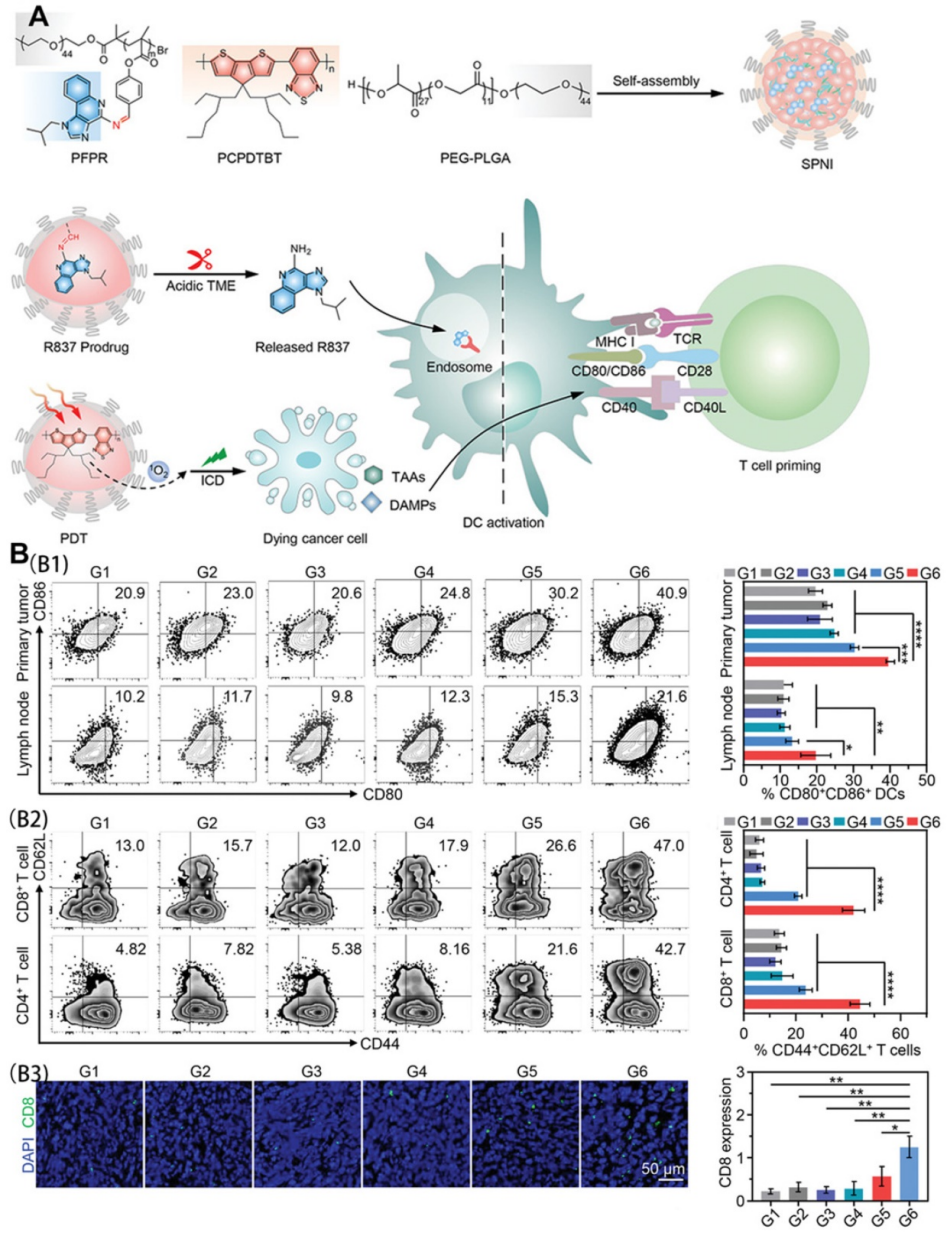
(A) Schematic illustration of semiconducting polymer nano-immunomodulator for NIR-induced photodynamic immunotherapy of cancer; (B) In vivo immune responses after NIR photodynamic immunotherapy. (B1) Representative flow cytometric plots and quantitative analysis of mature DCs (CD80^+^CD86^+^) in primary tumors and draining lymph nodes at day 3 after photoirradiation. (B2) Representative flow cytometric plots and quantitative analysis of central memory T cells (CD44^+^CD62L^+^) among CD4^+^ T cell subsets and CD8^+^ T cell subsets in primary tumors at day 24 after photoirradiation. (B3) Immunofluorescence staining images and quantitative analysis of CD8 of primary tumor sections from mice following various treatments. G1, PBS; G2, R837; G3, SPNC (-); G4, SPNI (-); G5, SPNC (+); G6, SPNI (+). Adapted with permission from 86, copyright 2022 John Wiley and Sons.

As shown in Figure 14A, SPNI not only mediates the production of a large amount of ROS but also significantly up-regulates CRT and HMGB1 in SPNI-treated cells under light radiation. The activation of DCs in tumor-draining lymph nodes was consistent with the results of DC activation in primary tumors. Analysis of the proportion of CD4^+^ T, CD8^+^ T, and central memory T cells (CD44^+^ CD62L^+^) showed that SPNI-mediated photodynamic immunotherapy can be used as a vaccine for cancer to stimulate strong anti-tumor T cell immunity, which helps to inhibit local and surrounding tumor growth (Figure 14B).

#### 4.1.2 DC vaccines

DCs are powerful APCs that effectively enhance the immune response. DCs are considered the central cell of the immune system because they provide a bridge between innate and acquired immune responses 87, 88. Recently, the progress of anti-cancer based on the combination of DC immunotherapy and PDT is in full swing 81, 82, 89. Rempolec et al. 82 constructed a PDT-based DC vaccine with a new photosensitizer OR141 (which can also be used as an ICD inducer) to enhance the priming effect of DCs to promote anti-tumor effects. The increased expression of costimulatory molecules, CD40, CD80, and CD86, indicated that the PDT-based DC vaccine successfully activates DC maturation. The prepared PDT-based DC vaccine conferred a higher survival rate to mice compared to those treated with CTLA-4 antibodies. This is further supported by the enhanced expression of CD8^+^ T cells, CD4^+^T cells, and INF-γ. Additionally, Zhang et al. 90 demonstrated that ALA (5-aminolevulinic acid)-PDT-induced tumor fragments could enhance the antigen presentation ability of DCs. Furthermore, in 2018, it was shown that ALA-PDT combined with a DC vaccine could induce an anti-tumor immune response 81. In this experiment, the DC vaccine based on PDT was shown to increase the number and activity of CD4^+^ T cells and CD8^+^ T cells in tumor tissue. In addition, in the PDT-DC vaccine group, the levels of INF-γ and IL-12 increased significantly in the peripheral blood, while the level of the immunosuppressive IL-10 decreased.

#### 4.1.3 PDT-motivated autologous tumor cell vaccine

In addition to immune cells, tumor cells can be used in combination with PDT for effective tumor vaccines. Fang et al. 89 prepared a PDT-motivated autologous tumor cell vaccine (P-ATV) by coating the copolymer of porphin E6 (PEI-CE6) on the surface of autologous tumor cells through electrostatic interaction. Subsequently, Fmoc-KCRGDK-phenylboronic acid (FK-PBA) solution and PC-cells dispersed in an alkaline solution were successively injected into the surgical site. FK-PBA can achieve enrichment in postoperative residual tumor sites by targeting sialic acid on the surface of tumor cells and gelation on demand, effectively encapsulating PEI-Ce6-coated autologous tumor cells (PC-Cell) to form PC-Cell@gel. The results showed that PC-Cell@gel combined with PDT could promote the maturation of APCs (mainly DCs) and inhibit regulatory T cells. More importantly, P-ATV was shown to effectively enhance CD8^+^ T cells specific for the new epitope, thus activating personalized immunity and significantly inhibiting tumor recurrence in melanoma and colon cancer mouse models.

### 4.2 PDT combined with immune checkpoint blockade

Although the current anti-tumor immunotherapy dominated by immune checkpoint blockades (ICBs) has achieved great clinical success, there remain many patients who do not respond well to ICB treatment 91. In-depth studies have shown that there is a lack of effective cytotoxic T cell infiltration in the TME of these patients, which is replaced by many immunosuppressive cells 92, 93. Photosensitizers can exert a photodynamic effect, generate ROS to induce tumor inflammation, improve tumor immunogenicity and promote intratumoral infiltration of cytotoxic T lymphocytes. Moreover, recent animal experiments have shown that PDT combined with ICB can produce synergistic therapy and activate a collective immune response 94-96. Therefore, the combination of PDT and ICB to trigger the anti-cancer immune response has been extensively studied.

**Figure 15:**
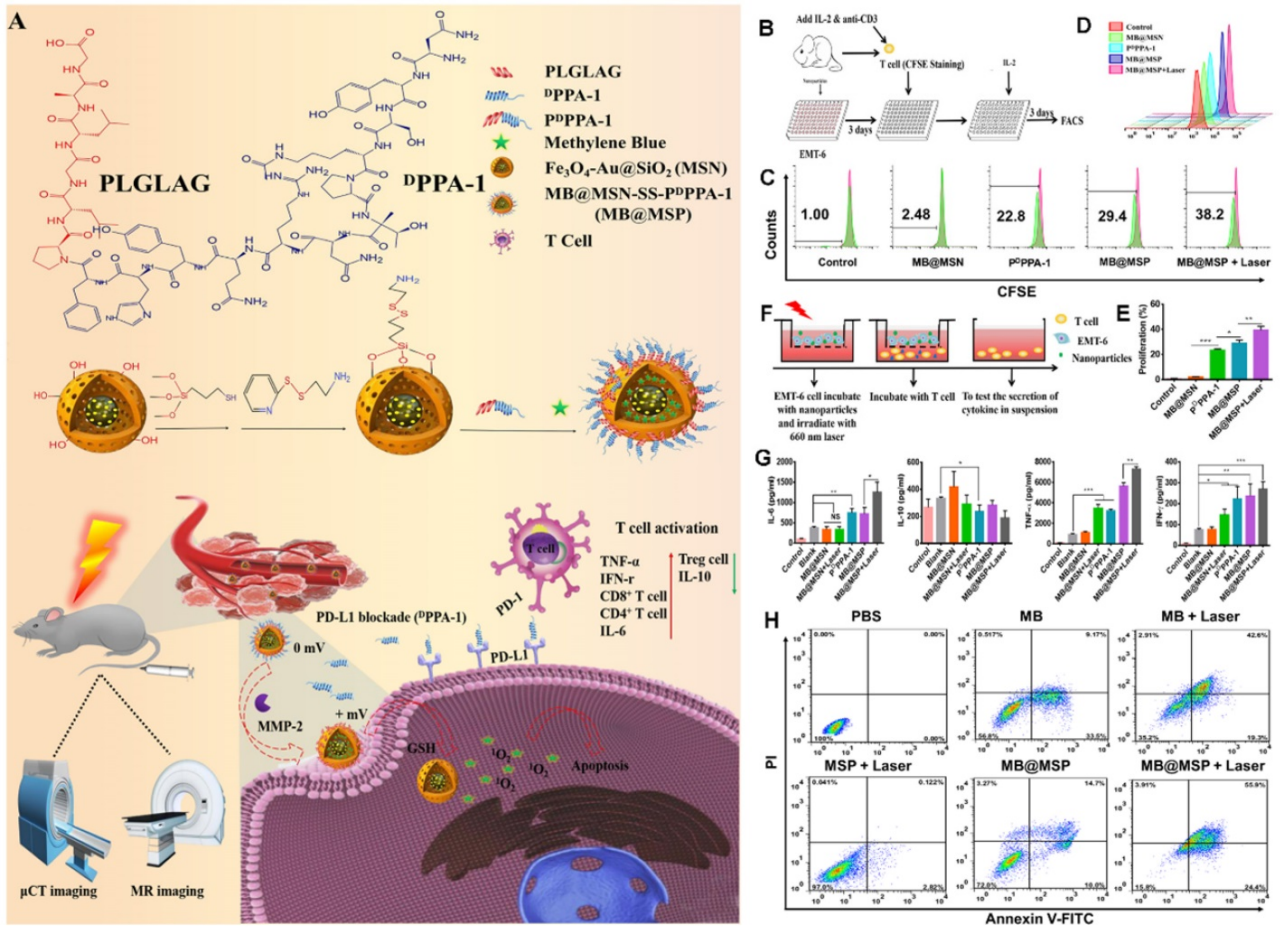
(A) Schematic illustration of the therapeutic mechanism of MB@MSP for PDT and immune therapy. (B) Schematic diagram of T cells and EMT-6 tumor cells co-culture system. (C, D) The proliferation rate of T cells was assessed after various treatments by flow cytometry analysis. (E) Quantification of the proliferation rate of T cells. (F) Schematic illustration of the in vitro Transwell co-cultured system, in which EMT- 6 tumor cells were pre-treated and then incubated with T cells. Tumor cells were placed in the upper chamber, and T cells were cultured in the lower chamber. Finally, cytokine secretion by T cells cultured system, measured by ELISA (G). (H) Flow cytometry data for Annexin V-FITC/PI-stained EMT-6 cells with different treatments, the lower left, lower right, upper right, and upper left quadrants represent the viable, early apoptotic, late apoptotic, and dead cells, respectively. Adapted with permission from 97, copyright 2021 Elsevier.

PD-1 is an important immunosuppressive transmembrane protein expressed on the surface of T cells. PD-L1 is the ligand of PD-1 and is induced by proinflammatory cytokines. In the TME, tumor cells can express PD-L1, which binds to PD-1 to inhibit T cell activation and cytokine production; thus, inhibition of the PD-1 pathway will enhance autoimmunity. As proposed in Figure 15A, Feng et al. 97 developed a novel immunomodulatory multifunctional nanoplatform (MB@MSP) based on a Fe_3_O_4_-Au core for photodynamic and ICB therapy guided by magnetic resonance (MR) and micro-computed tomography (μCT) imaging. In this nanoplatform, mesoporous silica nanoparticles with an Fe_3_O_4_-Au core can be used in nuclear MRI and μCT imaging to realize a non-invasive diagnosis of solid tumors and real-time dynamic monitoring of the drug delivery process. An anti-PD-L1 polypeptide (P^D^PPA-1) with matrix metalloproteinase-2 (MMP-2) activity was covalently linked to the surface of mesoporous silicon to prevent the leakage of the photosensitizer methylene blue (MB) during blood circulation. The cleavage of the P^D^PPA-1 peptide by highly expressed MMP-2 in the tumor matrix and the further destruction of the disulfide bond by a high concentration of glutathione in tumor cells eventually reduce the size of MB@MSP and reverse the charge. These changes promote the penetration and absorption of nanocarriers to tumors. The released P^D^PPA-1 peptide can block the immune checkpoint, create an environment conducive to the activation of cytotoxic T lymphocytes, and enhance tumor cell immunogenic death and tumor cell apoptosis caused by PDT, thus significantly improving the therapeutic effect (Figure 15A). In vitro cell experiments (Figures 15B-H) and in vivo results indicated that the proportion of T cells in the MB@MSP treatment group was significantly higher than that in the other groups. In other words, MB-mediated photodynamic and P^D^PPA-1 peptide-mediated PD-L1 blocking eliminate the systemic anti-tumor immune response by recruiting tumor-infiltrating CTLs. Additionally, the percentage of Treg cells in the MB@MSP treatment group decreased significantly, indicating that MB@MSP can regulate the immunosuppressive TME and further inhibit tumor metastasis and distal tumor development.

**Figure 16:**
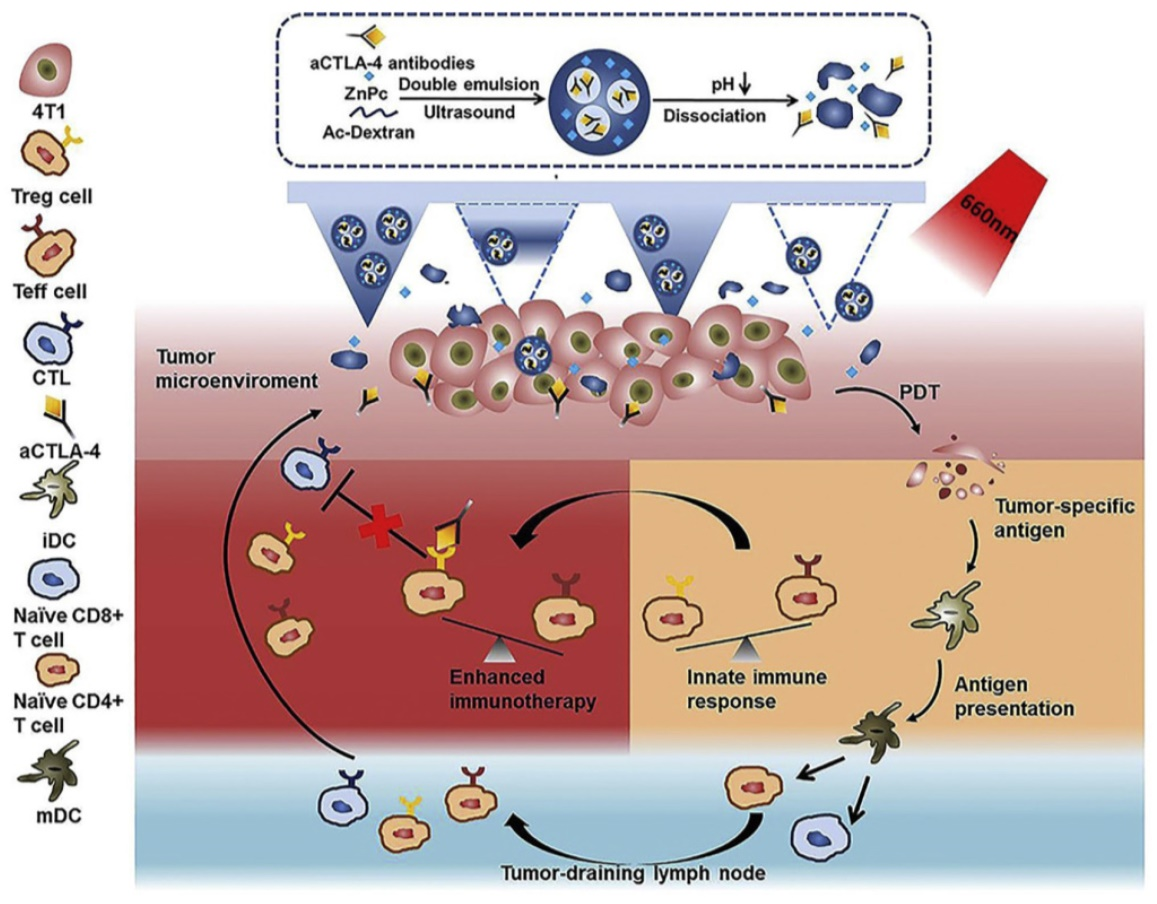
Scheme of the MN-assisted co-delivery system and possible mechanism of antitumor immune responses induced by MN-assisted PDT in combination with immune checkpoint blockade. Adapted with permission from 99, copyright 2020 Elsevier.

Another frequently exploited checkpoint is CTLA-4, which is expressed in Treg cells. The application of anti-CTLA-4 antibody combined with PDT has proven efficacy as an anti-cancer treatment that can restore immunity 98. Chen et al. 99 explored the photosensitizer zinc phthalocyanine (ZnPc) and α-CTLA-4 co-encapsulated dextran nanoparticles, namely, ZnPc/α-CTLA4@Ac-DEX NPs (Figure 16). The acidic TME can trigger ZnPc and the release of antibodies released during the degradation of Ac-Dexan by Ac-DEX nanoparticles. These nanoparticles with self-dissociation behavior were formed by an acid-degradable polymer matrix during the process of emulsion. In the 4T1 model of BALB/c mice, PS could produce cytotoxic ROS, damage tumor cells under 660 nm light irradiation, and combined with α-CTLA-4 to induce immunotherapy, but did not cause the systemic immune disorder.

Indoleamine 2,3-dioxygenase (IDO) is an intracellular enzyme overexpressed in tumor cells, which converts tryptophan (Trp) to kynurenine (Kyn), resulting in T cell anergy and promoting the differentiation of Tregs 100, severely impacting immune responses. Therefore, the combination of PDT and IDO inhibitors is considered a potential therapeutic strategy to improve anti-tumor immunity 101-104.

Recently, Huang et al. 104 reported a treatment method by coupling the IDO inhibitor NLG919 with the photosensitizer P_P_IX into liposomes to form P_P_IX-NLG@Lipo. After intravenous injection, nanoscaled P_P_IX-NLG@Lipo can preferentially gather in the tumor site and produce ROS, directly damaging primary tumor cells under light and leading to ICD, enhancing tumor immunogenicity. Meanwhile, P_P_IX-NLG@Lipo can interfere with IDO's regulation of Trp/Kyn metabolism, and ultimately reverse the immunosuppressive effect of the TME, turning “cold” tumors into “hot” tumors. Therefore, the combination of PDT and IDO blockers leads to the proliferation and infiltration of magnified CD8^+^ T cells, which inhibits not only primary and treated tumors but also distant tumors.

**Figure 17:**
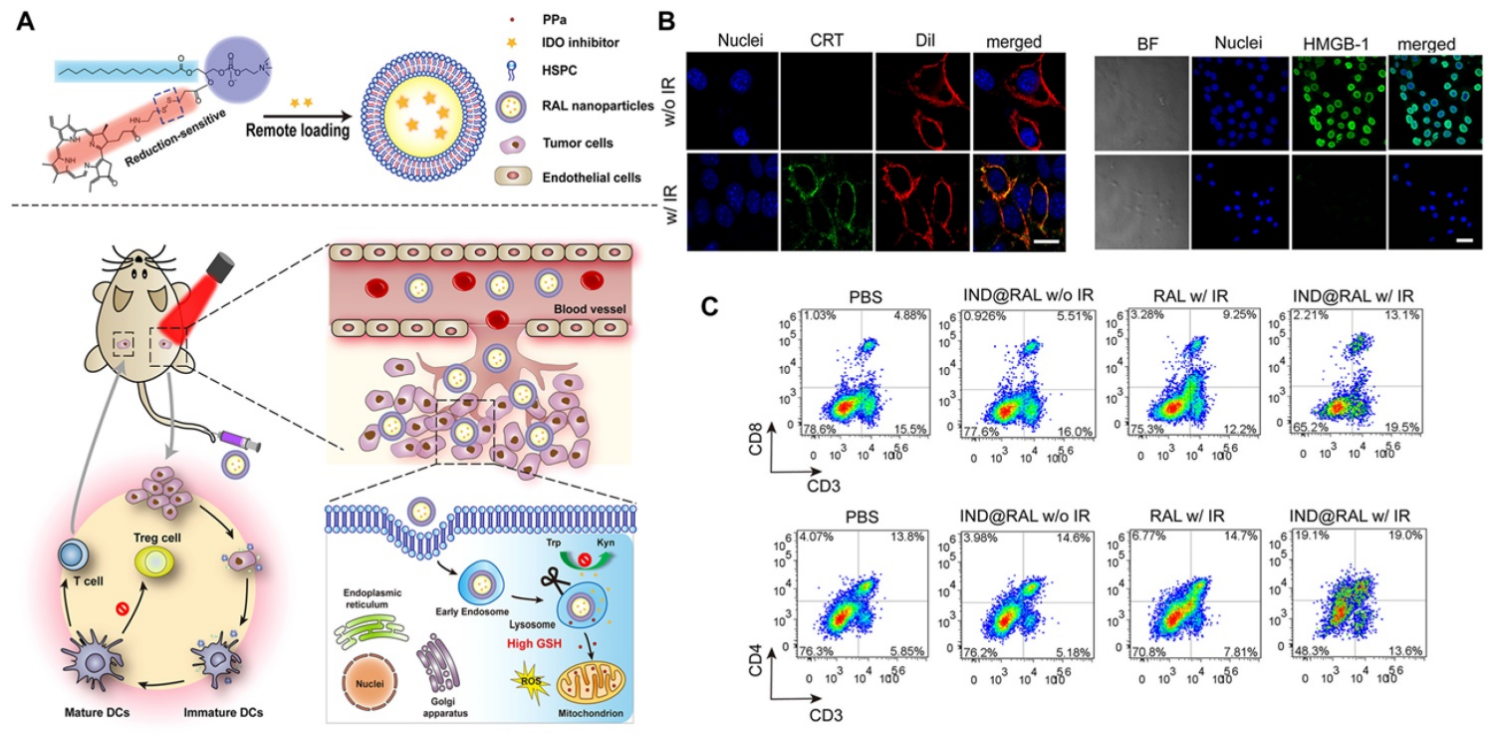
(A) Schematic Illustration of Combined PDT and Immunotherapy by IND@RAL for Combating Cancer. (B) Immunofluorescence imaging of CRT expression and HMGB1 release treated with RAL in the presence or absence of laser irradiation. (C) Flow cytometric analysis of CD3 and CD8, CD3 and CD4 positive cytotoxic T lymphocyte in spleen. Adapted with permission from 105, copyright 2019 American Chemical Society.

Liu et al. 105 further investigated the systemic immune effect induced by the combination of PDT and IDO to understand how to reverse the immunosuppressive TME. The researchers developed a redox-activated liposome called IND@RAL (Figure 17A), which could induce ICD and reverse the tumor suppressor microenvironment simultaneously by porphyrin-phospholipid coupling self-assembly and remote loading of IDO inhibitors (NLG8189) in the cavity. After intravenous injection, the IND@RAL prolonged the blood circulation of 4T1 tumor-bearing mice and promoted tumor accumulation. Following endocytosis of tumor cells, the nanovesicles activate fluorescence signaling and PDT activity exponentially in response to high intracellular glutathione levels, thus effectively inhibiting tumor growth. More importantly, redox-activated PDT induces intratumoral infiltration of CTLs by inducing the ICD of tumor cells, activating the innate immune system, and promoting antigen presentation (Figure 17B). Combined with IDO inhibitors, the anti-tumor immune response of the system will be further enhanced. As shown in Figure 17C, the percentage of CD3^+^ CD8^+^ T cells in the tumor tissue increased significantly after treatment, while the percentage of Treg cells decreased significantly, indicating that the combination of PDT and IDO inhibitors can systematically inhibit IDO activity and reverse the immunosuppressive TME. It activates innate immune cells to recognize necrotic tumor cells, deliver tumor-derived antigens to T cells, stimulates the infiltration of tumor-specific T cells, and thus has a strong killing effect on primary and distal tumors. In all, this strategy is expected to be used in PDT synergistic immunotherapy of metastatic cancer.

## 5. PDT combined with other therapies

Traditional cancer treatments, including chemotherapy and radiotherapy, as well as emerging cancer immunotherapies, including photothermal therapy, have potential and advantages in the field of cancer treatment. Therefore, a combination of multiple treatments is more effective than any single treatment in producing improved anti-cancer activity. In this section, the advantages of PDT-mediated immunization along with other treatments are summarized.

### 5.1 PDT combined with chemotherapy

Recent studies have found that some chemotherapeutic drugs, such as oxaliplatin 106, doxorubicin (DOX) 107, 108, and mitoxantrone (MTX) 109, can not only kill tumor cells but also cause ICD. Therefore, some researchers have combined chemotherapy with PDT to activate the T-cell immune response in the TME 107, 110-113.

**Figure 18:**
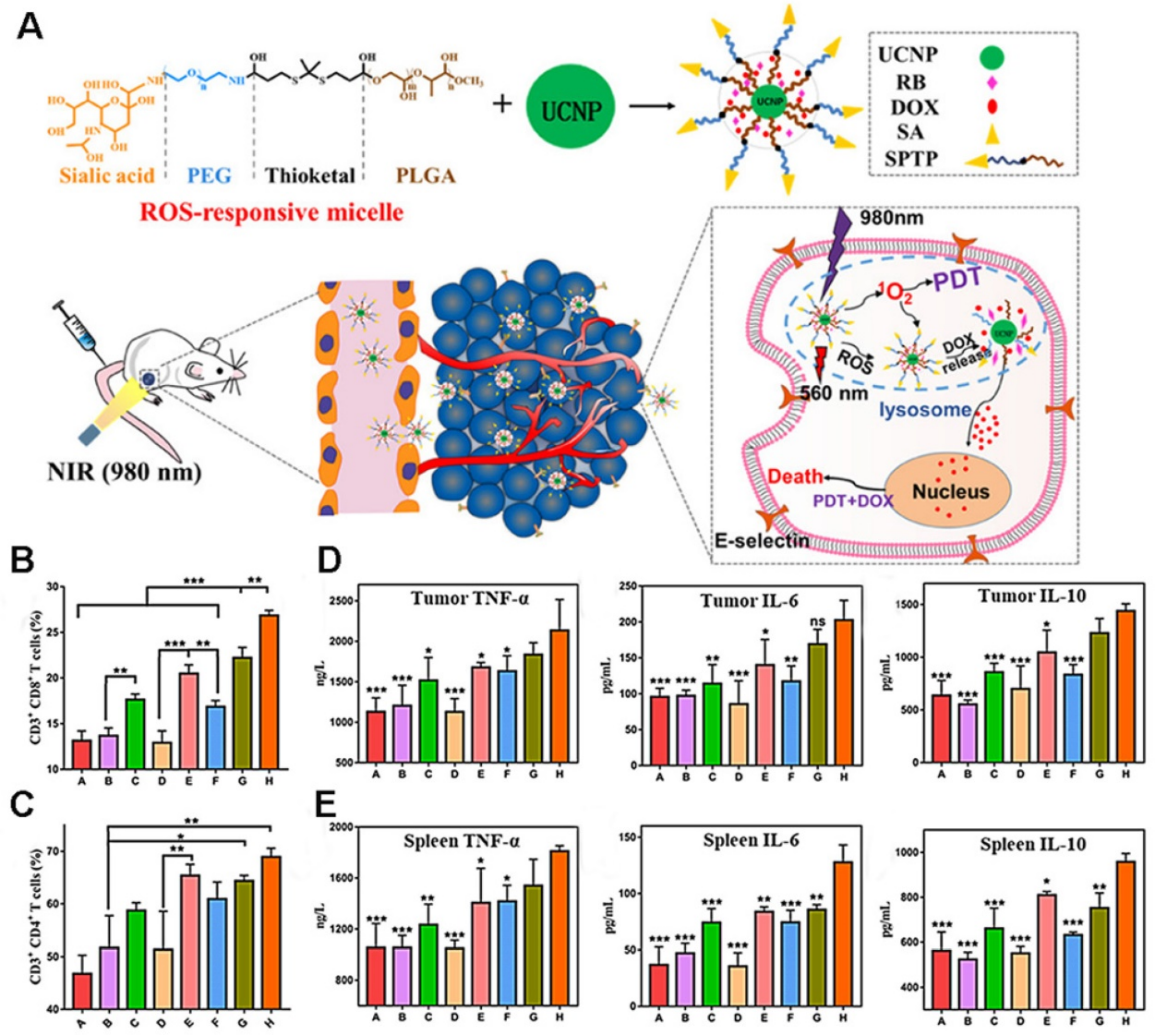
(A) Illustration of the anticancer application of SPTP@UCNP-Rb-DOX in TNBC murine model by cascade amplification of chemo-PDT with systematic anti-tumor immunity. Analysis of CD4^+^ T cells (B) and CD8^+^ T cells and (C) proliferation in spleen tissues collected from all mice groups. Quantitative analysis of TNF-α, IL-6, and IL-10 from tumor lysis solution (D) and (E) in spleen lysis solution by the Elisa kit. Adapted with permission from 107, copyright 2020 American Chemical Society.

As shown in Figure 18A, Jin et al. 107 co-encapsulated DOX and the photosensitizer rose bengal (RB) in ROS-responsive micelles based on upconversion nanoparticles (UCNP). The sialic acid (SA) on the surface of the micelle can specifically recognize the overexpressed E-selectin on the surface of tumor cells, leading to efficient targeting. After reaching the tumor, under the irradiation of near-infrared light, the photosensitizer RB receives energy to produce ^1^O_2_, which functions to kill tumor cells. Simultaneously, ROS production promotes the rupture of the micelle, releasing DOX, thereby demonstrating the synergistic effect of PDT and chemotherapy. The results showed that the prepared micellar particles initiated a large amount of 4T1 cell death (73.1%), and exhibited deep permeability and tumor accumulation. Moreover, the increase in CRT, ATP, and HMGB1 secretion levels indicated that PDT and DOX successfully induced the ICD and improved the anti-tumor immune response. The results of flow cytometry showed that the ratio of activated CD8^+^ T and CD4^+^ T cells was significantly increased in the micelle group, as were the levels of TNF-a, IL-10, and IL-6, suggesting that PDT combined with chemotherapy can activate the immune response in vivo (Figures 18B-E).

### 5.2 PDT combined with photothermal therapy

**Figure 19:**
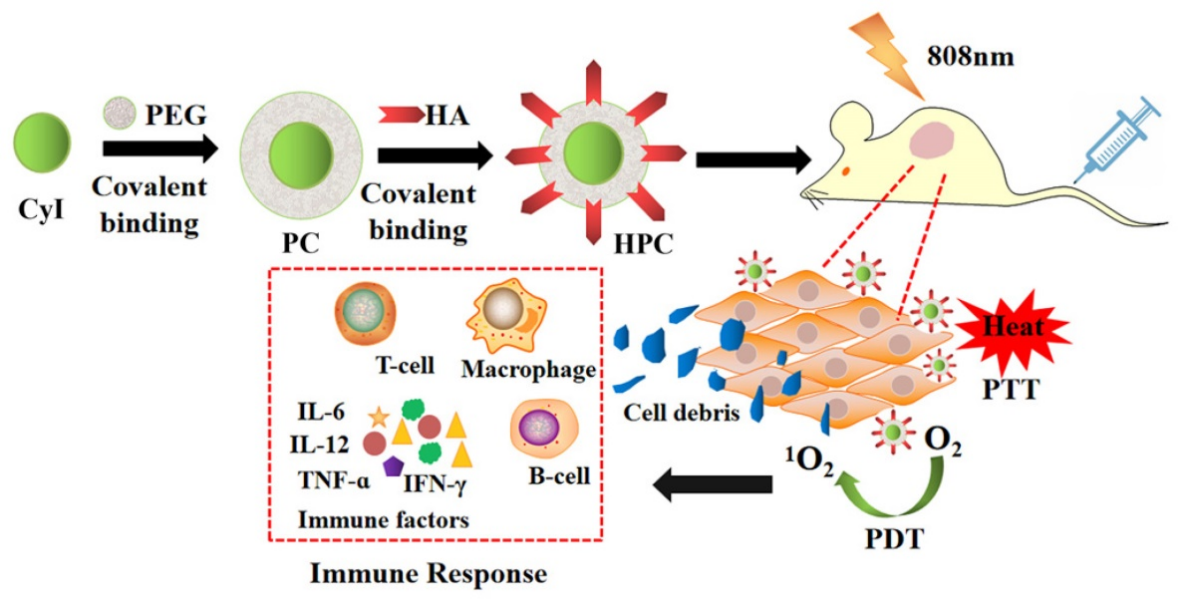
Schematic representation of the synthesis and phototherapeutic functions of the theranostic HPC. Adapted with permission from 6, copyright 2020, Royal Society of Chemistry.

Compared to PDT or PTT alone, synergistic phototherapy may conquer the extreme heterogeneity and complexity of refractory tumors and achieve better therapeutic effects. Recently, we synthesized a new iodinated-cyanine dye, named CyI, with stronger singlet oxygen (^1^O_2_) generating ability while maintaining excellent photothermal conversion efficiency and near-infrared fluorescence imaging compared to traditional cyanine dyes 6. Based on this, a novel targeted nanocarrier (HA-PEG-CyI) was designed by inducing the self-assembly of PEGylated CyI (iodinated-cyanine) by attaching a HA ligand on the surface 6 (Figure 19). HA is a biocompatible polysaccharide molecule that specifically binds to CD44 receptors overexpressed on the surface of tumor cells. Modifying HA with PEG-CyI is expected to increase the targeting ability of CyI. *In vivo* studies have shown that the novel targeted nanocarrier could effectively ablate tumors with a synergistic PDT/PTT effect, and further induce the release of tumor-related pro-inflammatory mediators (TNF-α, IFN-γ, IL-12, CTL), resulting in systemic anti-tumor immunity and secondary death of tumor cells.

**Figure 20:**
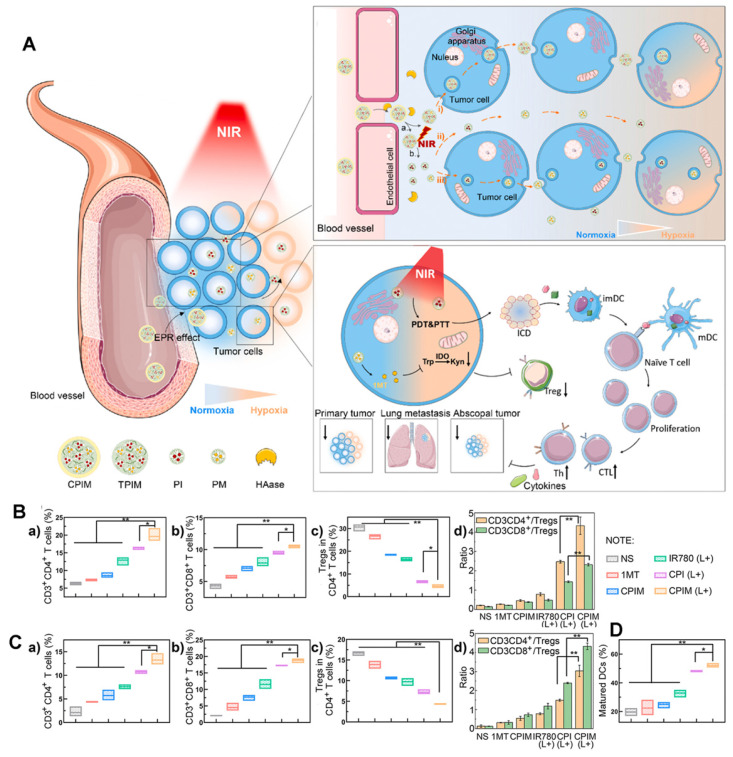
(A) Schematic illustration of the in vivo performance; (B) Analysis of percentages of Ths (CD3+ CD4+), CTLs (CD3+ CD8+), Tregs (CD4+ FOXP3+) and the ratio of Ths/Tregs and CTLs/Tregs in abscopal tumors (n=3); (C) Analysis of percentages of Ths (CD3+CD4+), CTLs (CD3+ CD8+), Tregs (CD4+ FOXP3+) and the ratio of Ths/Tregs and CTLs/Tregs in spleens (n=3); (D) Analysis of percentages of matured DCs (CD80^+^ CD86^+^) in tumor-draining lymph nodes (n=3). Adapted with permission from 114, copyright 2021 Elsevier.

The lack of deep infiltration of tumor tissue is a major limitation of drug therapy. In addition to active targeting, Yang et al. 114 for the first-time combined size-transforming and transcytosis strategies, which effectively enhanced passive diffusion and active transport to construct anti-tumor immune nanodrugs with efficient delivery efficiency (named CPIM) (Figure 20A). In terms of passive diffusion, cluster-bomb-like nanoplatforms (135 nm) release small drug-loaded “particles” (PAMAM loaded with IR780/1- methyltryptophan (1-MT), particle size < 10 nm) under the action of high concentrations of ROS in the TME, and promote the permeation and diffusion of IR780 and 1-MT in tumor tissue. Regarding active transport, the nanopreparation can be delivered by transcytosis, and in terms of anti-tumor activity, as shown in Figures 20B-D, CPIM significantly increased the penetration of CTL, with the highest proportion of CTL/Tregs and a significant increase in the DC maturity ratio. Moreover, the secretion of cytokines (including INF- γ, TNF- α, IL-6, IL-2) increased significantly. It was also confirmed that the nanopreparation can induce ICD, turn a “cold” immunosuppressive TME into a “hot” immunogenic TME, and inspire immunotherapy *in vivo*.

### 5.3 PDT combined with radiotherapy

**Figure 21:**
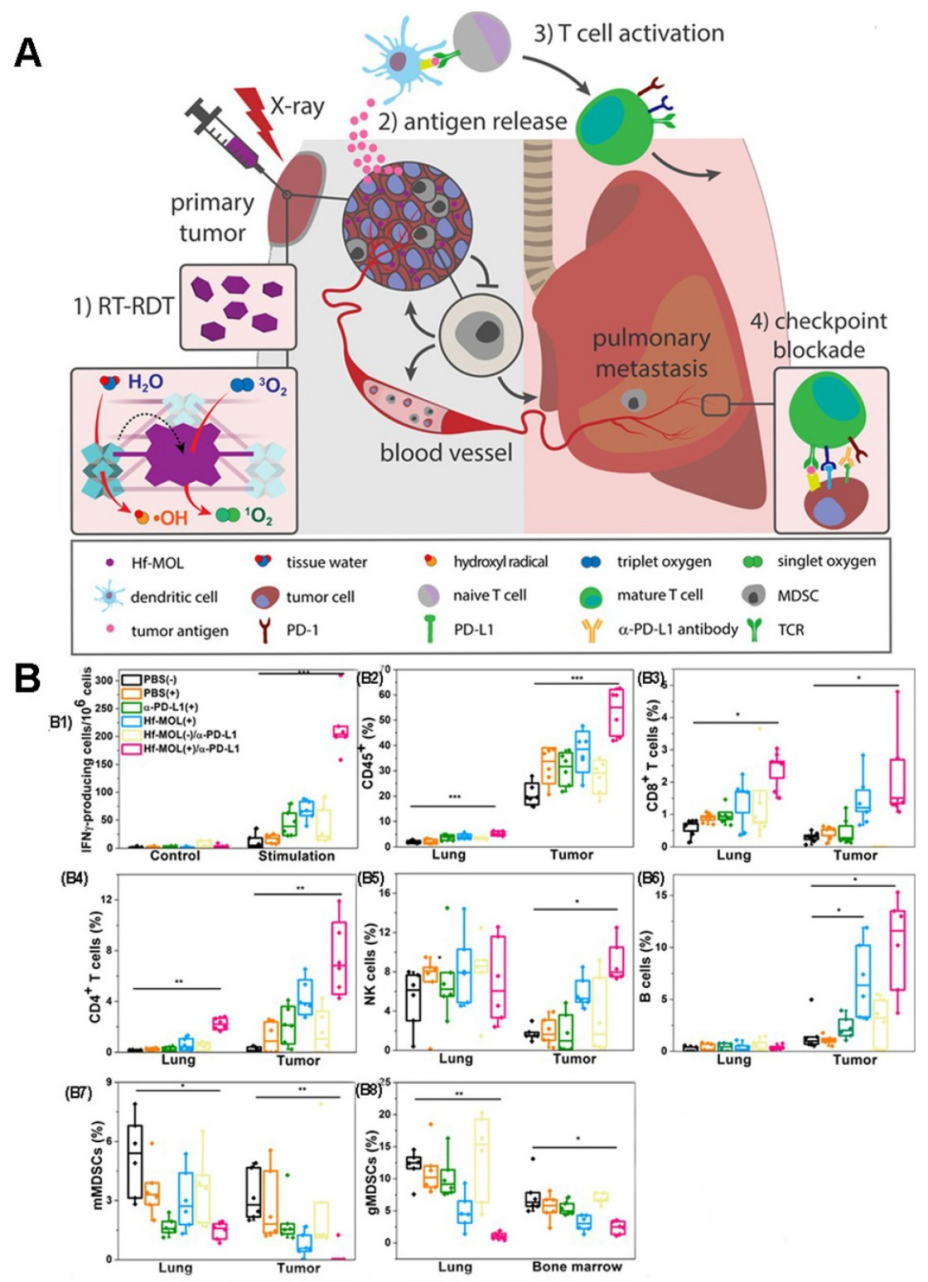
(A) Schematic illustration of combination therapy of PDT and X-rays. (B) Evaluation of anti-metastatic immunity in vivo. (B1) ELISPOT assay was performed to detect tumor-specific IFN-γ producing T cells. CD45^+^ (B2), CD8^+^ T cells(B3), CD4^+^ T cells(B4), NK cells(B5), B cells(B6), mMDSCs(B7) and gMDSCs (B8) with respect to the total tumor cells. Adapted with permission from 25, copyright 2019 Elsevier.

Previous studies have shown that Radiotherapy (RT) can induce systemic immune response 115, 116. Therefore, the combination of PDT and radiotherapy (known as Radio therapy-Radiodynamic therapy, RT-RDT) is also a method to improve the anti-tumor immune response 25, 117, 118. As proposed in Figure 21A, Lin et al 25 reported a new nanoscale metal-organic layer (nMOL), Hf-MOL, to effectively treated local tumors via radiotherapy-radiodynamic therapy (RT-RDT) with low-dose X-rays. When Hf-MOL is used in combination with immune checkpoint blockers, it can reverse metastatic tumor, improve tumor inflammation and activate systemic immunity by inducing immunogenic cell death (ICD).

The researchers evaluated the immunogenicity of Hf-MOL-mediated RT-RDT. By detecting the ATP, HMGB1 and CRT at the cellular level, the immunogenicity of Hf-MOL-mediated RT-RDT was significantly higher than that of nMOF-mediated radiotherapy. It was found that Hf-MOL mediated RT-RDT alone can effectively inhibit in situ tumor, but cannot achieve the inhibition of distal metastasis; while the combination of Hf-MOL mediated RT-RDT and α-PD-L1 mediated can not only greatly eliminate in situ tumor, but also effectively inhibit distal metastasis. After treatment, they analyzed the changes of tumor-infiltrating immune cells in the TME. Flow cytometry analysis showed that the expression of tumor-specific effector T cells, helper T cells, natural killer (NK) cells, and B cells up-regulated in tumors. When using ELISpot to detect IFN- γ in the spleen, the researchers found that the untreated tumor-bearing mice showed severe spleen enlargement compared with other non-lung-metastatic breast tumor models. Spleen size is generally regarded as a symbol of the immune response, and splenomegaly is considered a sign of acute immune toxicity. They described the splenocytes and detected significant upregulation of granulocytic MDSCs (gMDSCs) in the splenomegaly group. Only the gMDSC of HF-MOL(+)/α-PD-L1 treatment group decreased significantly, indicating that MOL(+)/α-PD-L1 could systematically consume gMDSC, and prevent abnormal splenic enlargement, which is a typical symptom of high metastatic tumor mice (Figure 21B).

## 6. Summary and prospects

Increasing evidence suggests that malignant tumors are not just a local disease, but instead represent a component of a complex adaptive system that involves inflammation, immunity, metabolism, and genetic disorders. Immunotherapy has been proven to have a great prospect in cancer treatment, but its low response rate and potential side effects remain major obstacles to its wide application in the clinic. Various combination therapies overcome these shortcomings while improving treatment outcomes. PDT, with minimal invasiveness, repeatability, no cumulative toxicity, excellent function, and cosmetic effect, is one such popular strategy, which can reduce the long-term incidence and improve the quality of life of patients. Most importantly, after the action of PDT, TAAs and related molecular damage patterns will be released, resulting in the death of immunogenic cells and the stimulation of the immune response. However, the weak immunity induced by PDT alone is insufficient to trigger a systemic immune response to cancer cells. Hence, in this study, we summarized and discussed recent advances and developments in the design of PDT-induced antitumor immunity in this review.

First, the complexity of the TME and the residue of cytokines and immune cells made it difficult to maximize the anti-tumor effect. Therefore, increasing attention has been paid to the role of PDT-mediated anti-tumor immunity in remodeling the immune TME, for example, using hyaluronidase targeting to destroy the ECM, VTP to break the physical barrier, or various oxygen supplement strategies to alleviate tumor hypoxia to improve the TME. Second, it has recently been found that the endoplasmic reticulum and mitochondria have immune potential as targets. Therefore, subcellular organelle and mitochondrial targeted PDT are also options for anti-tumor immunity. Additionally, PDT can be used as an in situ vaccine to promote anti-tumor effects. PDT can be combined with various additional immune stimuli (such as immune adjuvants and immune cells) to build a more powerful tumor vaccine. Finally, combination therapy—including immunotherapy, photothermal therapy, chemotherapy, and radiotherapy—is an effective strategy to improve the anti-tumor immune effect of PDT and transform “cold” tumors into “hot” tumors.

Although progress has been made, more efforts are needed to study photodynamic mediated anti-tumor immunity. For example, nanophotodynamic may be recognized and cleared by the reticuloendothelial system (RES) 119 or the mononuclear system (MPS) 120, thereby reducing the drug concentration at the tumor site; accordingly, appropriate biomaterials that mediate phototherapy should be developed. Although nanoparticles can be modified with PEG, PEG-modified nanoparticles can produce antibodies 121 and “accelerate blood clearance” 122 after the first injection (that is, PEG-encapsulated nanoparticles are quickly cleared by the liver at subsequent doses).

Modern immunotherapy brings new hope for tumor types with limited treatment methods and poor efficacy. From monotherapy to combination therapy, and onward to the discovery of new indications for drugs. In solid tumors, the greatest successes are obtained using ICB, with different mechanisms of action and clinical applications continuing to emerge. The shortcomings of immunotherapy also continue to emerge. Indeed, tumor cells can escape the killing effect of the innate and adaptive immune system, which makes ICB therapy ineffective. The inherent mechanisms of tumor immune escape include affecting the formation, presentation, and processing of new antigens through genetic and epigenetic changes, as well as altering signal transduction pathways that damage the role of cytotoxic T cells. External mechanisms include non-cancerous stromal or immune cells or other systemic effects that can cooperate with cancer cells to promote cancer cell growth and ICB resistance, such as host microbiota 93, 123. Importantly, several experimental studies have shown that the combination of phototherapy and ICB can promote the release of TAAs, thus triggering the tumor immune response and further promoting the blocking effect of ICB. However, there remains a lack of relevant clinical trials. Moreover, the differences in cytotoxicity and efficacy or treatment resistance caused by ICB are different from those of traditional chemotherapy or targeted therapy. At present, most clinical trials are limited to several tumor types, as well as lack of specific and effective targets 124.

Immunologic tolerance is a state of specific unresponsiveness when immunoreactive cells come into contact with antigenic substances. If T and B cells that respond specifically to antigens cannot be activated by antigen stimulation, they cannot produce specific immune effector cells to remember specific antibodies and thus cannot mount an effective immune response. There are many kinds of tumor-promoting immune cells in the TME, such as MDSCs, Tregs, and tumor-associated macrophages (TAMs). MDSCs secrete ROS, NO to inactivate T cells, and TGF β and IL-10 to activate Tregs 125, 126. Moreover, ROS can induce the accumulation of SUMO-specific protease 3 (SENP3), which is related to the anti-tumor immunosuppression of Treg to T cells 127. Additionally, ROS can stimulate the transformation of TAMs 128 and induce Tregs 129 to inhibit the expression of CTLs. Therefore, ROS are not only a metabolic by-product of biological cells but also an important intracellular signal molecule involved in tumor immune response and tolerance, although the specific mechanism requires further study.

Finally, due to the inconsistent results of different models of primary tumors and the difficulty of combining biomarkers, the effect and efficiency of the comprehensive treatment are not always comparable. As such, more comprehensive treatment research could be conducive to the development of successful photo-immunotherapy drugs.

## Figures and Tables

**Table 1:** Summary of different strategies for PDT-induced antitumor immunity.

Category	Strategies	Ref
Reprogramming tumor microenvironment	Disrupting the ECM	37
	Angiogenesis blockade	43
	Delivering O_2_ to hypoxic tumors	22, 52
	Producing O_2_ at the hypoxic tumor	54, 56, 59
Subcellular targeted photodynamic therapy	Endoplasmic reticulum targeted PDT	64, 65
	Mitochondrial-targeted PDT	75, 76
PDT combine with immunotherapy	Adjuvant	85, 86
	DC vaccine	82,90
	PDT-motivated autologous tumor cell vaccine (P-ATV)	89
	PDT combined with ICB	97, 99
	PDT combined with IDO	104, 105
Other combination therapies	PDT combined with chemotherapy	107
	PDT combined with PTT	6, 114
	PDT combined with radiotherapy	25

**Table 2:** Summary of different formulations for PDT-induced antitumor immunity by reprogramming TME. (↑= upregulation, ↓= downregulation)

Formulation	Therapeutic agents	Immune expression	Ref
DEX-HAase	Ce6, anti-PDL1	TAAs, IFN-γ, TNF-α, CD8^+^, mDC↑ HIF-1α, M2 macrophage↓	37
TOOKAD	WST11, anti-PD-1/anti-PD-L1	TAAs, CD4^+^, CD8^+^ ↑ Treg, PD-L1↓	43
FS@PMPt	Ce6, PFC	TAAs, mDC, CD4^+^, CD8^+^, TGF-β, IFN-γ↑ HIF-1α, Treg↓	22
PF_11_DG	DiD	TAAs, mDC, NK, CD8^+^↑ MDSC, HIF-1α↓	52
FA-L@MD@CAT	MBDP, CAT	TAAs, mDC, CTL, M1 macrophage↑, M2, Treg, Hypoxia↓	54
MnO_2_@chitosan-CyI	MnO_2_, CyI	TAAs, CTL, IFN-γ, TNF-α↑ M2 macrophage, Hypoxia↓	56
CeO_2_@MSNs@IR780/Met	CeO_2_, IR780	TAAs, mDC, CD4^+^, CD8^+^↑ HIF-1α, PD-L1↓	59

**Table 3:** Summary of different formulations for PDT-induced antitumor immunity by combination therapy. (↑= upregulation, ↓= downregulation)

Formulation	Therapeutic agents	Immune expression	Ref
PCN-ACF-CpG@HA	CpG, H_2_TCPP	TAAs, IFN-γ, TNF-α, CTL, mDC↑	85
Semiconducting polymer nano-immunomodulator (SPNI)	R837, PCPDTBT	TAAs, CD4^+^, CD8^+^, mDC, central memory T cell↑	86
DC vaccine	OR414	TAAs, CD4^+^, CD8^+^, mDC↑	82
ALA-PDT-DC cancer vaccine	ALA	TAAs, IFN-γ, TNF-α, CTL, mDC↑ IL-10↓	90
PC-Cell@gel	PEI-CE6, FK-PBA	TAAs, IFN-γ, TNF-α, CTL, mDC↑	89
MB@MSP	MB, anti-PD-L1 polypeptide	TAAs, IFN-γ, TNF-α, CTL, mDC, IL-6↑ Treg, IL-10↓	97
ZnPc/α-CTLA4@Ac-DEX	ZnPc, α-CTLA4	TAAs, CD4^+^, CD8^+^, mDC↑ Treg, CTLA4↓	99
PpIX-NLG@ Lipo	NLG919, PPIX	TAAs, CD8^+^, Trp↑ Kyn↓	104
IND@RAL	NLG8189, RAL	TAAs, mDC, CD4^+^, CD8^+^↑ Kyn, Treg↓	105
